# Identification of a pituitary ERα-activated enhancer triggering the expression of *Nr5a1*, the earliest gonadotrope lineage-specific transcription factor

**DOI:** 10.1186/s13072-019-0291-8

**Published:** 2019-08-07

**Authors:** Vincent Pacini, Florence Petit, Bruno Querat, Jean-Noël Laverriere, Joëlle Cohen-Tannoudji, David L’hôte

**Affiliations:** Sorbonne Paris Cité, Université Paris-Diderot, CNRS UMR 8251, INSERM U1133, Biologie Fonctionnelle et Adaptative, Physiologie de l’axe gonadotrope, Paris, France

**Keywords:** Gonadotrope specification, Enhancer, Epigenetic, *Nr5a1*, Estrogen receptor

## Abstract

**Background:**

Gonadotrope lineage differentiation is a stepwise process taking place during pituitary development. The early step of gonadotrope lineage specification is characterized by the expression of the *Nr5a1* transcription factor, a crucial factor for gonadotrope cell fate determination. Abnormalities affecting *Nr5a1* expression lead to hypogonadotropic hypogonadism and infertility. Although significant knowledge has been gained on the signaling and transcriptional events controlling gonadotrope differentiation, epigenetic mechanisms regulating *Nr5a1* expression during early gonadotrope lineage specification are still poorly understood.

**Results:**

Using ATAC chromatin accessibility analyses on three cell lines recapitulating gradual stages of gonadotrope differentiation and in vivo on developing pituitaries, we demonstrate that a yet undescribed enhancer is transiently recruited during gonadotrope specification. Using CRISPR/Cas9, we show that this enhancer is mandatory for the emergence of *Nr5a1* during gonadotrope specification. Furthermore, we identify a highly conserved estrogen-binding element and demonstrate that the enhancer activation is dependent upon estrogen acting through ERα. Lastly, we provide evidence that binding of ERα is crucial for chromatin remodeling of *Nr5a1* enhancer and promoter, leading to RNA polymerase recruitment and transcription.

**Conclusion:**

This study identifies the earliest regulatory sequence involved in gonadotrope lineage specification and highlights the key epigenetic role played by ERα in this differentiation process.

**Electronic supplementary material:**

The online version of this article (10.1186/s13072-019-0291-8) contains supplementary material, which is available to authorized users.

## Background

*Nr5a1* gene (also called *Sf*-*1* or *Ad4BP*) is a transcription factor (TF) belonging to the nuclear receptor superfamily. In mammals, *Nr5a1* is expressed notably in testes, adrenal glands, ventromedial hypothalamic nucleus (VMH) and anterior pituitary gland where it participates in embryonic cell differentiation and adult function [1]. The anterior pituitary is composed of six hormone-secreting cell types, i.e., corticotrope, melanotrope, somatotrope, lactotrope, thyrotrope and gonadotrope cells, originating from common precursor stem cells of the Rathke’s pouch [2]. Several TFs are known to promote pituitary stem cells differentiation into a specific endocrine lineage: POU1F1 is mandatory for the thyrotrope, somatotrope and lactotrope lineages [3], TBX19 for the corticotrope [4], PAX7 for the melanotrope [5] and *Nr5a1* for the gonadotrope lineage [6]. During gonadotrope cells specification, *Nr5a1* is the earliest specific marker gene known to be expressed [6] initiating transcription of key genes such as *Gnrhr* (GnRH receptor gene) and *Lhb* (β-subunit of the gonadotropin LH gene). As a consequence, mutations in the human *Nr5a1* gene [7] and *Nr5a1* knockout in mice lead to gonadotrope deficiency [6].

*Nr5a1* expression depends on tissue-specific *cis*-regulatory elements. Two main promoters, 1A and 1G, have been characterized. The 1G promoter is the predominantly activated promoter in the pituitary [8]. However, it is not able, alone, to initiate *Nr5a1* expression [9]. Additional distal enhancers are required for tissue-specific transcription. Four specific enhancers have been identified that control expression of *Nr5a1* in the VMH [10], fetal adrenal glands [11], fetal Leydig cells [12] or gonadotrope cells [13]. The gonadotrope enhancer has been suggested to be implicated in *Nr5a1* expression in gonadotropes from mouse embryonic day 13.5 (E13.5) onwards [13]. In a previous work [14], we characterized the epigenetic marks decorating *cis*-regulatory regions of *Nr5a1*. We used a set of three cell lines recapitulating three stages of gonadotrope differentiation. The αT1–1 cells are likely derived from E12.5 common precursor cells from which originate thyrotrope and gonadotrope lineages. These cells do not express *Nr5a1* yet. The αT3–1 cells are likely derived from cells engaged in a gonadotrope cell fate at E13.5 and express some of the gonadotrope-specific genes including *Nr5a1.* The LβT2 cells are likely derived from mature gonadotrope cells and express all the known marker genes [15–17]. We observed that although *Nr5a1* is already expressed in αT3–1 cells, the epigenetic marks on the gonadotrope enhancer indicate that it is repressed, suggesting that it does not regulate *Nr5a1* expression at early steps of gonadotrope specification.

In this work, using functional genomic in vitro and in vivo approaches, we demonstrated that *Nr5a1* expression is triggered by another early activated enhancer at the emergence of gonadotrope lineage. We showed that this enhancer is activated by the estrogen pathway through ERα leading to P300 histone acetyltransferase recruitment. We also demonstrated that ERα protects the enhancer from inhibition by DNA methylation and chromatin compaction. Finally, we showed that the enhancer interacts with the *Nr5a1* pituitary promoter and increases histone acetylation, RNA polymerase recruitment and *Nr5a1* transcription. Activation of this enhancer is thus the earliest known mechanism implicated in gonadotrope cell specification.

## Results

### Differential chromatin accessibility in *Nr5a1* locus during gonadotrope specification

**Fig. 1:**
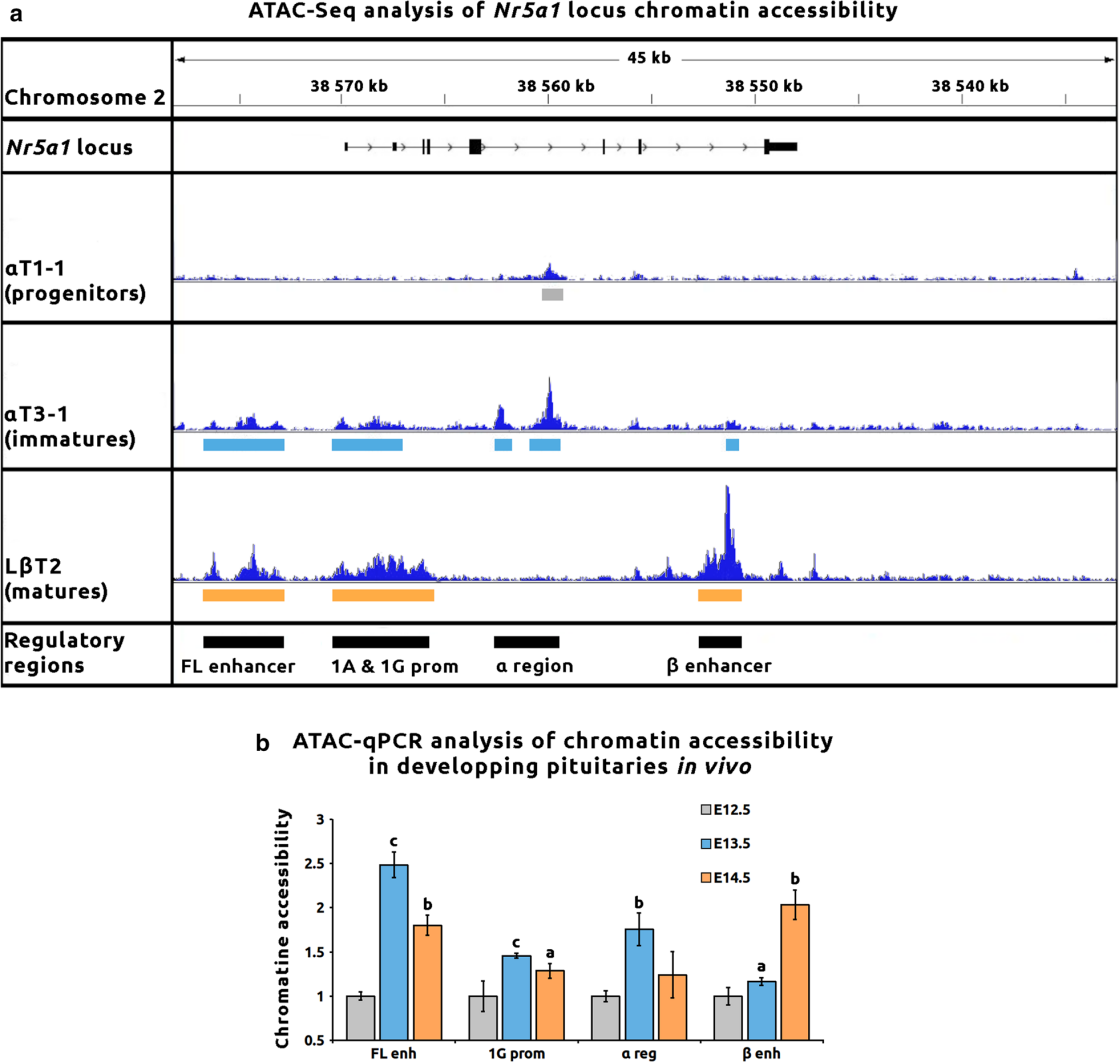
Differential chromatin accessibility in the *Nr5a1* locus during gonadotrope specification. **a** The *Nr5a1* locus displays differential local chromatin accessibility depending on gonadotrope differentiation stage in vitro. Chromatin accessibility was investigated by assay for transposase-accessible chromatin with high-throughput sequencing (ATAC-seq) in αT1–1, αT3–1 and LβT2 gonadotrope cell lines. ATAC-seq tracks are shown for 45 Kb of the *Nr5a1* locus. Accessible chromatin regions identified from ATAC-seq results are shown for each cell line under each track (respectively, in gray, blue and yellow). In the last lane are summarized in black the potential regulatory regions, based on accessible chromatin sequences identified in at least one cell line. **b** *Nr5a1* potential regulatory regions show differential chromatin accessibility depending on gonadotrope differentiation stage in vivo. ATAC assay followed by real-time PCR quantification (ATAC-qPCR) was performed on pituitary cells dispersed from developing glands of E12.5, E13.5 and E14.5 mouse embryos. Quantitative PCR was performed using sets of primers targeting the potential *cis*-regulatory regions. Raw qPCR data were normalized to control region (see “Materials and Methods”) and to E12.5 stage of development. At this stage, *Nr5a1* gene is not expressed and the locus is expected to be in a closed chromatin conformation. Results are the mean ± SEM of three independent experiments. Change in chromatin accessibility between E12.5 and E13.5 or E12.5 and E14.5 embryonic stages is compared using a Mann–Whitney test. Significant difference with E12.5 stage: “a” *p* < 0.05; “b” *p* < 0.01; “c” *p* < 0.001

We performed an assay for transposase-accessible chromatin with high-throughput sequencing analysis (ATAC-seq) in αT1–1, αT3–1 and LβT2 cell lines, allowing the identification of new potential *cis*-regulatory sequences (Fig. 1a and Additional file 1). We observed massive changes in chromatin accessibility in the three cell lines with about 20,000 specific accessible regions per line. Genomic regions associated with genes known to be expressed in the three cell lines, such as C*ga* or *Isl1* promoters, were open in all cell lines. Regions associated with *Gnrhr* promoter were accessible in both αT3–1 and LβT2, whereas those associated with *Lhb* promoter were only found in LβT2 cells (Additional file 1). Chromatin accessibility is thus consistent with the maturation stage of these cellular models.

We then analyzed chromatin accessibility of the *Nr5a1* locus. In αT1–1, consistent with the absence of *Nr5a1* expression, very few genomic regions were accessible in the *Nr5a1* locus. In αT3–1, several open chromatin regions could be observed and among them two had already been described: the 1G promoter, in agreement with *Nr5a1* expression and, more surprisingly, the fetal Leydig enhancer (FL enhancer). This enhancer has been formerly described to be active specifically in the fetal testis [12]. The gonadotrope enhancer (named hereafter β enhancer) showed only very limited chromatin accessibility in αT3–1 cells, in agreement with our previous observations [14]. The chromatin was also accessible for a previously undescribed region encompassing two very close peaks in intron 4 (peak-1: mm9 chr2:38,559,896–38,560,286 and peak-2: mm9 chr2:38,562,187–38,562,583), named hereafter the α region. This region exhibited only limited chromatin accessibility in αT1–1 cells. In LβT2 cells, the 1G promoter as well as the FL and β enhancers all showed accessible chromatin conformation. In contrast, the chromatin of the α region was no more accessible.

These results strongly suggested that the *Nr5a1* locus displays dynamic chromatin accessibility during gonadotrope lineage differentiation. In order to investigate whether the same dynamics could be observed in vivo, we set up an ATAC assay followed by qPCR on developing pituitaries of mouse embryos (Fig. 1b). We observed that the 1G promoter was significantly open at E13.5 and E14.5 compared to E12.5, in agreement with the *Nr5a1* expression dynamics during mouse pituitary development [6]. Chromatin at the α region was strongly and transiently accessible at E13.5. While chromatin at the β enhancer displayed a very limited accessibility at E13.5, it was fully open at E14.5. Finally, the FL enhancer was open from E13.5 onwards. Differential chromatin accessibility in *Nr5a1* locus during gonadotrope specification was thus validated in developing embryos in vivo.

### Discovery of an undescribed early enhancer in *Nr5a1* locus

**Fig. 2:**
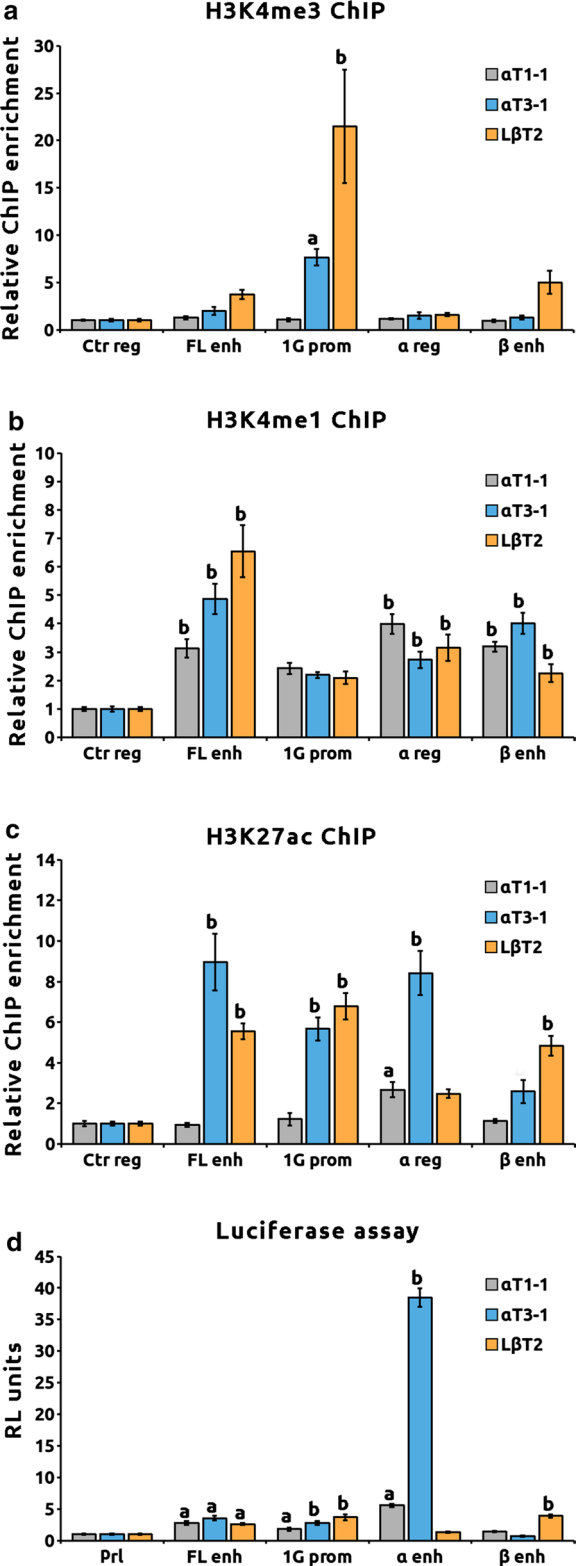
Discovery of an undescribed early enhancer of *Nr5a1* gene. **a**, **b** and **c** *Nr5a1*-accessible chromatin regions harbor differential epigenetic marks of active enhancers depending on gonadotrope stage of differentiation. Epigenetic modifications of histone H3 associated with ATAC-seq open regions were investigated using chromatin immunoprecipitation assays in αT1–1, αT3–1 and LβT2 cell lines. Analyzed marks were lysine 4 tri- and monomethylation H3K4me3 (**a**) and H4K4me1 (**b**), specific of promoters and enhancers, respectively, and lysine 27 acetylation H3K27ac (**c**), enriched on active *cis*-regulatory sequences. Quantitative PCR was performed using sets of primers targeting ATAC-seq open regions. Raw qPCR data were normalized to input. The final results were expressed as fold over the control region. ANOVA followed by Dunnett’s multiple comparison tests was performed independently for each cell line and each histone modification. Results are the mean ± SEM of six independent experiments. Significant difference with the control region: “a” *p* < 0.05; “b” *p* < 0.01. **d** *Nr5a1*-accessible chromatin regions display differential *cis*-regulatory activity depending on gonadotrope differentiation stage. αT1–1, αT3–1 and LβT2 cells were transiently transfected with the potential *cis*-regulatory regions cloned in a pGL3b luciferase reporter system containing a minimal prolactin promoter (Pluc–Prl). Relative luciferase activity was measured as indicated in “Materials and Methods.” ANOVA followed by Dunnett’s multiple comparison tests was performed independently for each cell line. Results are normalized to control Pluc–Prl plasmid and are the mean ± SEM of six independent experiments. Significant difference with the control construct: “a” *p* < 0.05; “b” *p* < 0.01

In order to further characterize regions with chromatin accessibility, we studied in the three cell lines the deposition of H3K4me3, H3K4me1 and H3K27ac histone marks, specific for promoters, enhancers and active elements, respectively (Fig. 2a–c). Only the 1G promoter was significantly enriched with H3K4me3 in αT3–1 and LβT2 cells (Fig. 2a). All other regions had significant H3K4me1 enrichment at these three stages of gonadotrope maturation, indicating that they all are potential enhancers (Fig. 2b). Concerning deposition of the active chromatin mark (Fig. 2c), the 1G promoter and FL enhancer were enriched in H3K27ac only in αT3–1 and LβT2 cells. While the β enhancer was decorated with H3K27ac only in LβT2, the α potential enhancer was significantly decorated with H3K27ac in αT1–1 and αT3–1 cells (Fig. 2c, fold enrichment of 2.6 ± 0.4, *p* < 0.05 and 8.4 ± 1.0, *p* < 0.01, respectively).

Potential *cis*-regulatory activity was then studied in luciferase reporter system (Fig. 2d). We observed that the FL enhancer and 1G promoter were active in the three cell lines. A significant *cis*-regulatory activity of the α enhancer peak-2 could be already observed in αT1–1 that was strongly increased in αT3–1 (fold induction of 5.6 ± 0.8 and 38.5 ± 1.4 over control Pluc–Prl, *p* < 0.01 in αT1–1 and αT3–1, respectively) but lost in LβT2 cells. As peak-1 did not show any regulatory activity in the αT3–1 cell line (Additional file 2A), subsequent experiments were performed on the peak-2 only and α enhancer will thereafter refer to this peak. The β enhancer displayed a significant *cis*-regulatory activity only in LβT2 cells (fold induction of 3.8 ± 0.2, *p* < 0.01). Altogether these results suggest that: (i) the β enhancer is active only during the terminal maturation stage; (ii) the FL enhancer might also be recruited in gonadotropes; (iii) the α region is a genuine gonadotrope enhancer potentially activated in precursor and immature cells.

### The α enhancer regulates *Nr5a1* expression specifically in immature gonadotropes

**Fig. 3:**
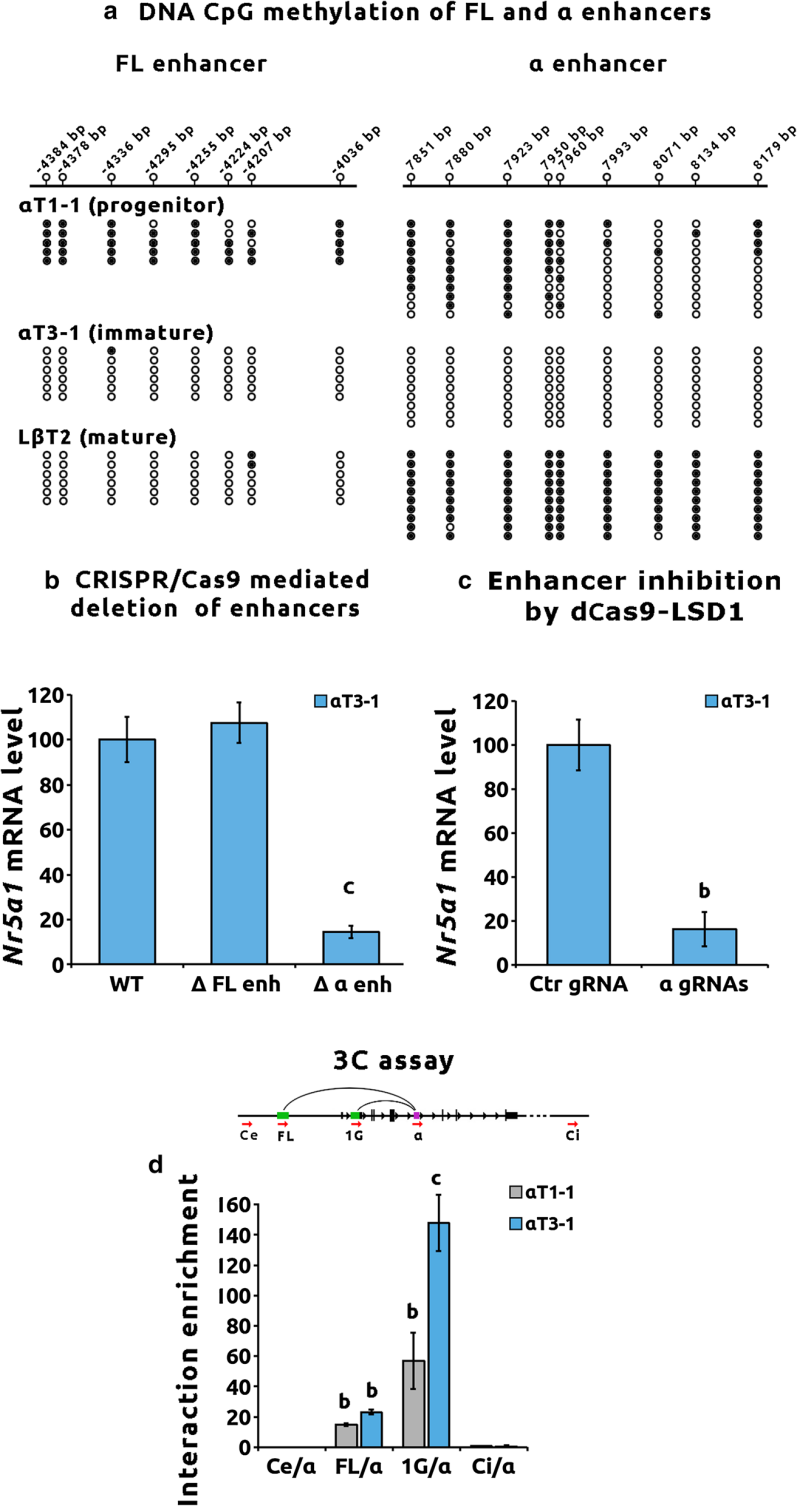
The α enhancer regulates *Nr5a1* expression specifically in immature gonadotropes. **a** The α enhancer exhibits different DNA CpG methylation status according to gonadotrope differentiation stage. Genomic DNA of αT1–1, αT3–1 and LβT2 cells was extracted and bisulfited. The FL enhancer and α enhancer-bisulfited sequences were amplified and cloned. A minimum of five clones per cell line was sequenced. Top: Schematic representation of enhancer sequences with location of CpGs (open-circle lollipops). Numbering is relative to *Nr5a1* 1A promoter TSS. The state of CpG methylation for each cell line, methylated (black circles) or unmethylated (open circles), is indicated below. **b** The regulation of *Nr5a1* expression is dependent on the α enhancer sequence in immature αT3–1 cells. Deletion of genomic sequence of the FL and α enhancers was carried out in αT3–1 cells using CRISPR/Cas9 and two independent specific guide RNA (gRNA) couples flanking each enhancer sequence: α gRNA1–gRNA3 or α gRNA2–gRNA4 for the α enhancer and FL gRNA1–gRNA3 or FL gRNA2–gRNA4 for the FL enhancer as described in Additional file 7. Untargeting control gRNA was used as control. For each gRNA couples, three independent homozygous clones were tested for *Nr5a1* expression by RT-qPCR. *Nr5a1* expression level was normalized to *Gapdh*. Data are the normalized mean ± SEM of six independent experiments. WT and Δ FL enh αT3–1 or Δ α enh αT3–1 clones were compared with ANOVA followed by Dunnett’s multiple comparison tests. Significant difference with WT: “c” *p* < 0.001. **c** The α enhancer is a functional enhancer of *Nr5a1* in immature αT3–1 cells. The α enhancer was decommissioned in αT3–1 cells using CRISPR/dCas9 fused with the lysine-specific histone demethylase LSD1 coding sequence (dCas9–LSD1). The dCas9–LSD1 was targeted to the α enhancer genomic sequence using α gRNA1–gRNA3 or α gRNA2–gRNA4 gRNA couples. Untargeting control gRNA (Ctr gRNA) was used as control. The 25% highly transfected cells were retrieved using cytometry cell sorting and tested for *Nr5a1* expression by RT-qPCR. *Nr5a1* expression level was normalized to *Gapdh*. Data are the normalized mean ± SEM of three independent experiments and are compared to control untargeting gRNA using Student’s *t* test “b” *p* < 0.01. **d** The α enhancer interacts with *Nr5a1* pituitary promoter in progenitor and immature cells. Top: Quantitative chromatin conformation capture (**c**) assay was carried out in αT1–1 and αT3–1 cells. Chimeric DNA fragments were detected using a fixed forward primer targeting the α enhancer and several forward primers targeting regions upstream, inside or downstream from the 1G pituitary promoter sequence as shown in the schematic diagram of *Nr5a1* structure. Primers positions are indicated with red arrows for Ce (external to locus control region), FL (fetal Leydig enhancer), 1G (1G promoter), α (α enhancer) and Ci (internal to locus control region). Exons are indicated as dark bars, regulatory regions as green bars and α enhancer as a purple bar. Numbering is relative to 1A promoter TSS. Bottom: Histograms showing qPCR measurements of chimeric fragments in 3C library. Raw qPCR data were normalized to input and to Ci/α chimeric DNA used as a control of non-specific ligation events. RP23 225F7 bacterial artificial chromosome (BAC) was used to create template enabling quantitative measurement of chimeric regions in the 3C library. Data are the mean ± SEM of four independent experiments and were analyzed with ANOVA followed by Dunnett’s multiple comparison tests. Significant difference with Ci/α: “b” *p* < 0.01; “c” *p* < 0.001, nd: not detected

CpGs DNA methylation of the active enhancers was investigated by bisulfite DNA sequencing. As shown in Fig. 3a, CpGs of the α enhancer were mostly hypermethylated in progenitors, fully demethylated in immature and methylated again in mature cells. The FL enhancer was hypermethylated in αT1–1 and unmethylated in αT3–1 and LβT2 cells. Thus, both DNA methylation and histone decoration show that the FL enhancer is totally inactive in progenitors and active in immature and mature cells. In contrast, the α enhancer is in a bivalent state in progenitors, exhibiting both active and inactive marks, active in immature and totally silent in mature gonadotropes.

To further confirm the regulatory role of the α enhancer in *Nr5a1* expression, the peak-2 genomic sequence was excised from αT3–1 genome by CRISPR/Cas9. Excision was also performed for the FL enhancer. Deletion of the FL enhancer did not alter *Nr5a1* expression in αT3–1, whereas deletion of α enhancer sequence led to a strong reduction in expression as revealed by a 85% decrease in *Nr5a1* mRNA level (Fig. 3b, *p* < 0.001). This drastic reduction was observed in three independent clones for two independent gRNA couples, ruling out potential gRNA off target effects. (The genomic sequences of deleted clones along with gRNAs position are shown in Additional file 2B.) In order to inactivate this enhancer without altering the DNA sequence, we targeted dCas9–LSD1, a lysine-specific histone demethylase to the α enhancer. In αT3–1 cells, this led to an 80% decrease in *Nr5a1* mRNA level as compared with control gRNA (Fig. 3c, *p* < 0.01). Decommissioning the α enhancer in LβT2 cells did not impair *Nr5a1* expression, while targeting LSD1 to the β enhancer strongly reduced it (fold decrease of about 90%, Additional file 3A). The transient α enhancer activation is thus mandatory for *Nr5a1* expression specifically in immature gonadotropes. Chromatin interaction between the α enhancer and 1G promoter was investigated in αT1–1 and αT3–1 using quantitative chromatin conformation capture (3C) assay (Fig. 3d). We also tested potential interactions with control regions, external (Ce) and internal (Ci) to the *Nr5a1* locus as well as the FL enhancer. No interaction between the α enhancer and the upstream region (Ce/α) could be detected in the two cell lines. In contrast, in αT3–1 cells (and to a lesser extent in the αT1–1), the α enhancer interacts significantly with the 1G promoter as well as with the FL enhancer (Fig. 3d).

### ERα controls *cis*-regulatory activity of the α enhancer

**Fig. 4:**
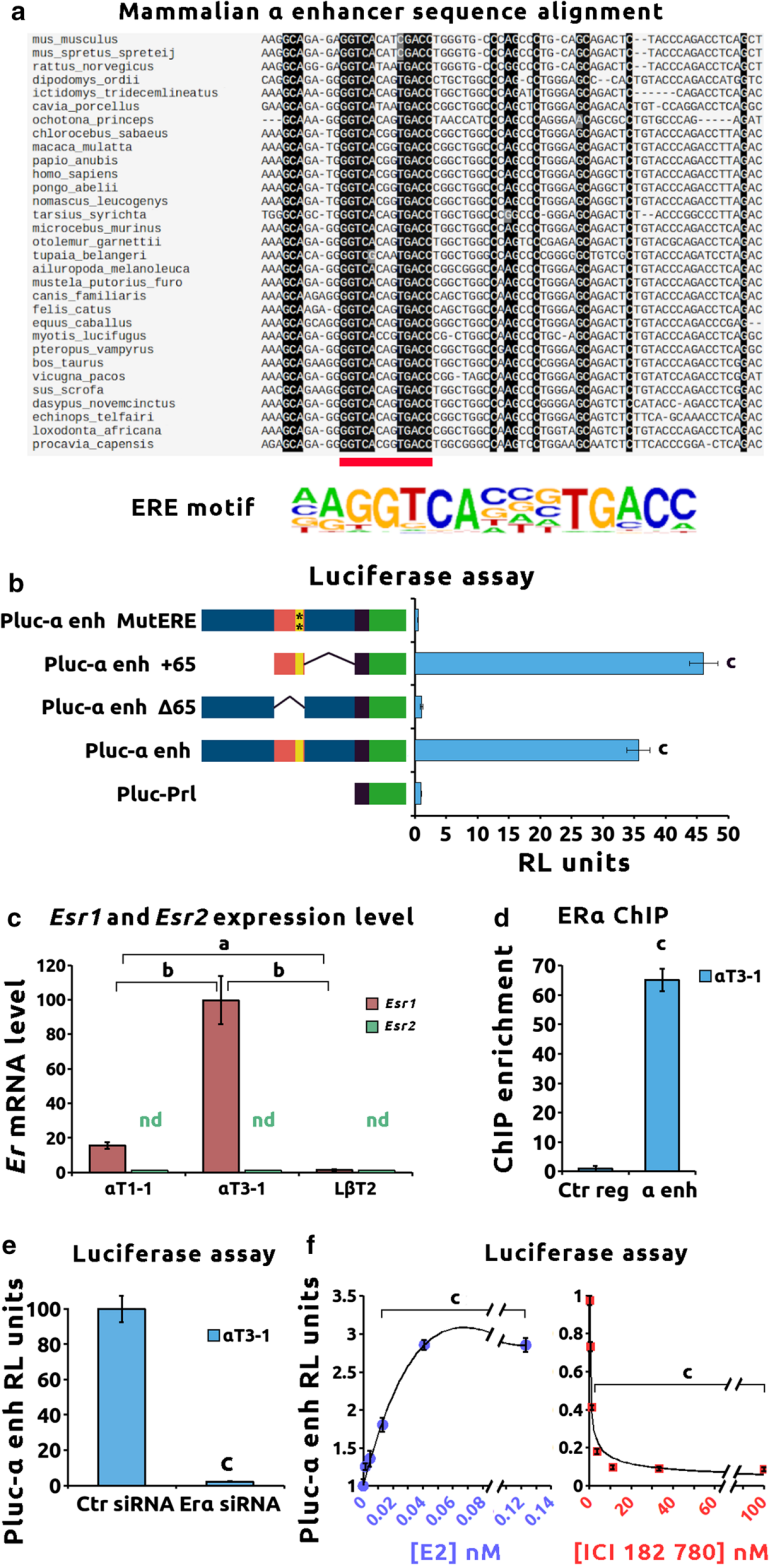
ERα controls the *cis*-regulatory activity of the α enhancer. **a** A 65-bp core sequence of the α enhancer is conserved across mammalian genomes. The α enhancer genomic sequences of 31 mammalian species were retrieved on Ensembl Web site and aligned using Clustal Omega software. A 65-bp core sequence displays clear conservation among the different species. In this core sequence, a perfect match with the canonical ERE-binding site was observed (red bar and ERE-binding motif). **b** The ERE-binding site in the 65-bp core sequence is essential for α enhancer *cis*-regulatory activity in immature αT3–1 cells. αT3–1 cells were transiently transfected in complete steroid-containing culture medium with Pluc constructs containing either a minimal prolactin promoter (Pluc–Prl) used as control, a full-length α enhancer (Pluc–α enh), a truncated α enhancer (Pluc–α enh Δ65, harboring a deletion of the 65-bp core sequence), a reduced α enhancer (Pluc–α enh +65, containing only the 65-bp core sequence) or a mutated α enhancer (Pluc–α enh MutERE with the following mutation in the ERE-binding site: GATCAATGTGATC). Prl minimal promoter is represented in dark followed by luciferase coding sequence shown in green, α enhancer is represented in blue, containing the 65-bp core sequence in orange, and the ERE-binding site in yellow. ERE mutation is indicated with asterisks. Relative luciferase activity was measured as indicated in “Materials and Methods.” Results were normalized to Pluc–Prl used as control and are the mean ± SEM of six independent experiments. Comparisons with control were performed with ANOVA followed by Dunnett’s multiple comparison tests. Significant difference with Pluc–Prl control: “c” *p* < 0.001. **c** *Erα* but not *Erβ* is expressed in immature αT3–1 cells. Total RNA from αT1–1, αT3–1 and LβT2 cells was extracted and reverse transcribed. The mRNA levels of *Erα* and *Erβ* were quantified as indicated in “Materials and Methods.” Results are normalized to *Gapdh* and are the mean ± SEM of four independent experiments. ANOVA followed by Bonferroni’s post hoc comparisons test was performed to compare *Erα* expression in the different cell lines. Significant difference: “a” *p* < 0.05 and “b” *p* < 0.01. Nd: not detected. **d** Endogenous ERα binds to the α enhancer chromatin in immature αT3–1 cells. ERα binding on α enhancer chromatin was investigated using ChIP assays in the αT3–1 cell line using the anti-estrogen receptor alpha ChIP-grade antibody (abcam ab32063). Quantitative PCR was performed using primers targeting the α enhancer sequence (α enh). Raw qPCR data were normalized to input. The final results were expressed as fold over the control region (Ctr region). Results are the mean ± SEM of four independent experiments. Significant difference with the control region using Student’s *t*-test: “c” *p* < 0.001. **e** The *cis*-regulatory activity of α enhancer is strictly dependent on *Erα* expression level. αT3–1 cells were transiently co-transfected with control (Pluc–Prl) or full-length α enhancer (Pluc–α enh) constructs and with scramble or *Erα* SiRNA. Relative luciferase activity was measured as indicated in “Materials and Methods.” Results were normalized to control Pluc–Prl plasmid and are the mean ± SEM of six independent experiments. Significant difference with the scramble SiRNA using Student’s *t* test “c” *p* < 0.001. **f** ERα agonist and antagonist modulate α enhancer *cis*-regulatory activity. αT3–1 cells were transiently transfected with control (Pluc–Prl) or full-length α enhancer (Pluc–α enh) constructs. Transfected cells were treated with either vehicle, E2 or ICI 182,780 at the indicated concentrations. Relative luciferase activity was measured as indicated in “Materials and Methods.” Results were normalized for control Pluc–Prl and are the mean ± SEM of six independent experiments. ANOVA followed by Dunnett’s multiple comparison tests was performed to compare drugs at different concentrations against vehicle. Significant difference with the vehicle: “c” *p* < 0.001

In order to understand the mechanisms regulating α enhancer activity, genomic sequence conservation of the α enhancer peak-2 was analyzed across mammals (Fig. 4a). A 65-bp core sequence (mm9 chr2:38,560,050–38,560,114) is conserved including a 13-bp stretch showing more than 60% of conservation. According to the *cis*BP online library [18], this element corresponds to a perfect ERE motif. No other conserved binding site could be identified in the rest of the core sequence (Additional file 2C).

In order to test the involvement of this 65-bp core sequence in the α enhancer *cis*-regulatory activity, truncated sequences were tested by luciferase reporter assay in αT3–1 cells maintained in complete steroid-containing medium (Fig. 4b). The full-length α enhancer construct (Pluc–α enh) displayed a significant *cis*-regulatory activity as compared to the minimal prolactin promoter (Pluc–Prl) used as control. The 65-bp core sequence (Pluc–α enh +65) showed similar activity as Pluc–α enh. Furthermore, deletion of the 65-bp core sequence (Pluc–α enh Δ65) or mutation of the potential ERE motif (Pluc–α enh MutERE) completely abolished *cis*-regulatory activity in αT3–1 cells. This demonstrates that the 65-bp core conserved sequence is sufficient alone to drive α enhancer activity and that the ERE motif is crucial for this activity.

Both estrogen receptors α (ERα) and β (ERβ) bind to ERE motif. *Esr1* and *Esr2* mRNAs were quantified by RT-qPCR (Fig. 4c). While *Esr2* transcripts were undetectable, *Esr1* was expressed in αT1–1 and αT3–1 cells, with a fivefold higher expression level in αT3–1 cells (Fig. 4c, *p* < 0.01). In mature LβT2 cells, *Esr1* mRNA could hardly be detected.

ChIP using an anti-ERα antibody was then performed in αT1–1 and αT3–1 cells. ERα was strongly enriched on the α enhancer as compared to the control region in αT3–1 cells (Fig. 4d, *p* < 0.001). Binding of ERα to the α enhancer was also observed to a lesser extent in αT1–1 cells (Additional file 4A, *p* < 0.01).

To further analyze the role of ERα in the regulation of α enhancer activity, *Esr1* expression was knocked down using *Esr1* SiRNA. Efficiency in ERα decrease was validated by western blot (Additional file 5A). Specific knockdown of ERα abolished α enhancer *cis*-regulatory activity in αT3–1 cells (Fig. 4e, *p* < 0.001). A significant decrease in α enhancer activity was also observed in αT1–1 cells (Additional file 4C).

In order to test ligand dependency, α enhancer activity was measured in the presence of 17β-estradiol (E2) or the widely used antagonist ICI 182,780 in the αT3–1 cells. While E2 dose-dependently activated α enhancer *cis*-regulatory activity, ER inhibition led to a dose-dependent repression (Fig. 4f). A similar repression could be observed using the ERα-specific antagonist, MPP dihydrochloride (Additional file 5B).

To confirm α enhancer dependency to both ERα and E2, activities of the WT- and ERE-mutated α enhancers were assessed in the presence of E2 combined with ERα over-expression in LβT2 cells. We observed that α enhancer activity can be significantly induced by ERα over-expression alone (fold induction compared to basal of 19.7 ± 3.5, *p* < 0.001, Additional file 3B) and further increased by ERα over-expression combined with E2 treatment (fold induction compared to basal of 65.1 ± 5.4, *p* < 0.001, Additional file 3B). Mutation of the ERE abolished ERα and E2 effects on α enhancer *cis*-regulatory activity.

In order to investigate whether ERα is sufficient to activate the endogenous α enhancer in mature gonadotrope cells, ERα was over-expressed in LβT2 cells and the α enhancer chromatin accessibility was assessed by ATAC-qPCR. We observed that over-expression of ERα is sufficient to significantly increase α enhancer chromatin accessibility in mature gonadotropes (fold increase in chromatin accessibility of 2.2 ± 0.4, *p* < 0.01 Additional file 3C).

These results altogether demonstrate that the α enhancer is active only in immature gonadotrope cells and that this activity is regulated by ERα and E2.

### ERα controls *Nr5a1* expression through epigenetic regulation of the α enhancer and 1G promoter

**Fig. 5:**
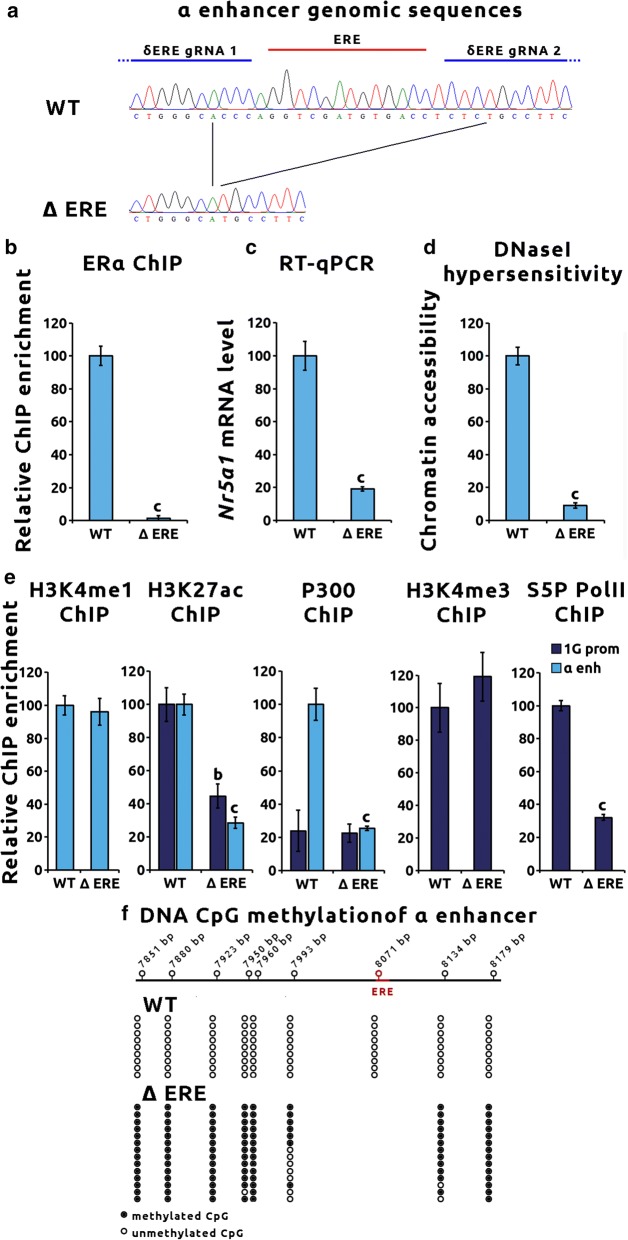
ERα controls *Nr5a1* expression through epigenetic regulation of the α enhancer. **a** Deletion of the α enhancer ERE-binding site using CRISPR/Cas9 in immature αT3–1 cells. Deletion of ERE genomic sequence in α enhancer was carried out in αT3–1 cells using CRISPR/Cas9 and a couple of specific gRNA flanking the ERE sequence. Untargeting gRNA was used as control. Two independent homozygous clones for both deletion and for control gRNA were sequenced. The genomic sequences of WT and deleted ERE (Δ ERE) αT3–1 clones are shown along with the ERE motif and the δERE–gRNA positions. **b** Deletion of the α enhancer ERE prevents ERα binding to the enhancer chromatin in immature αT3–1 cells. ERα binding on α enhancer chromatin was investigated using ChIP assays in WT and Δ ERE αT3–1 clones. Quantitative PCR was performed using primers targeting the α enhancer genomic sequence. Raw qPCR data were normalized to input. The final results were expressed as fold over the control region. Results are the mean ± SEM of six independent experiments. Significant difference with the control region was analyzed using Student’s *t*-test: “c” *p* < 0.001. **c** An intact ERα-binding site in the α enhancer is essential for *Nr5a1* expression in immature αT3–1 cells. *Nr5a1* expression in WT and Δ ERE αT3–1 cells was measured by RT-qPCR. *Nr5a1* expression level was normalized to *Gapdh*. Data are the normalized mean ± SEM of six independent experiments. Significant difference with the WT using Student’s *t*-test: “c” *p* < 0.001. **d** Abolition of the ERα binding site in α enhancer leads to a strong reduction in the α enhancer chromatin accessibility in immature αT3–1 cells. The α enhancer chromatin accessibility was investigated using DNAse I hypersensitivity (DNase I HS) assay in WT and Δ ERE αT3–1 clones. Quantitative PCR was performed using primers targeting the α enhancer genomic sequence. Raw qPCR data were normalized to input. The final results were expressed as fold over the control region. Results are the mean ± SEM of four independent experiments. Significant difference with the WT using Student’s *t*-test: “c” *p* < 0.001. **e** Abolition of the ERα-binding site in the α enhancer prevents active chromatin marks deposition on the α enhancer and 1G promoter in immature αT3–1 cells. Monomethylation of Lys4 (H3K4me1), acetylation of Lys27 on histone H3 (H3K27ac) and trimethylation of Lys4 (H3K4me3) epigenetic modifications as well as binding of P300 and serine 5-phosphorylated RNA polymerase II (S5P Pol II) to the α enhancer and/or 1G promoter sequences were studied using ChIP assays in WT and Δ ERE αT3–1 clones. Quantitative PCR was performed using sets of primers targeting the α enhancer or 1G promoter sequence. Raw qPCR data were normalized to input. The final results are expressed as fold over the control region. Results are the mean ± SEM of six independent experiments. Significant difference between WT and Δ ERE αT3–1 clones for each mark and each *cis*-regulatory element was studied using Student’s *t*-test: “b” *p* < 0.01; “c” *p* < 0.001. **f** Abolition of the ERα-binding site in the α enhancer leads to CpG hypermethylation of the α enhancer chromatin in immature αT3–1 cells. Genomic DNA of WT and Δ ERE αT3–1 clones was extracted and bisulfited. The α enhancer-bisulfited sequences were amplified and cloned. A minimum of nine clones per cell line was sequenced. Top: schematic representation of the α enhancer sequence with location of CpG (open-circle lollipops) and of the ERE site. Below is indicated the state of CpG methylation for each cell line, methylated (black circles) or unmethylated (open circles)

To specifically inhibit ERα binding to the α enhancer, the ERE motif of α enhancer genomic sequence was excised using CRISPR/Cas9 in immature gonadotropes. Two αT3–1 clones (ΔERE) bearing homozygous deletion of this ERE were retrieved. As expected, the deletion encompassed the ERE plus four additional bases at each side (Fig. 5a). ChIP assay showed that ERα enrichment on α enhancer was dramatically decreased in ΔERE clones as compared to WT (99% decrease *p* < 0.001) (Fig. 5b).

*Nr5a1* expression level was then quantified in ΔERE clones. Compared to WT clones, *Nr5a1* expression was decreased by 85% in ΔERE clones (Fig. 5c, *p* < 0.001).

To further characterize α enhancer regulation by ERα, epigenetic remodeling at the α enhancer and 1G promoter was then investigated in ΔERE clones. DNase I hypersensitivity assay revealed that excision of ERE-binding site led to a 90% decrease in α enhancer chromatin accessibility (Fig. 5d, *p* < 0.001). Comparison of histone mark decoration of α enhancer between ΔERE and WT clones revealed that deletion of ERE did not impact H3K4me1 deposition on the α enhancer (Fig. 5e). However, a 75% drop in H3K27ac enrichment could be observed (Fig. 5e, *p* < 0.001).

Binding of the P300 histone acetyltransferase to the α enhancer was then investigated by ChIP using anti-P300 antibody. P300 was significantly enriched on the α enhancer in αT3–1 and to a lesser extent in αT1–1 cells (Additional file 4B, *p* < 0.01). Deletion of ERE led to an 80% decrease in P300 recruitment in αT3–1 cells (Fig. 5e, *p* < 0.001). CpGs DNA methylation was analyzed in ΔERE compared with WT clones (Fig. 5g). Inhibition of ERα binding led to a hypermethylation of the α enhancer in immature gonadotropes.

Analysis of the 1G promoter epigenetics revealed that α enhancer repression did not affect H3K4me3 mark deposition. It, however, decreased H3K27ac enrichment on the 1G promoter (60% decrease *p* < 0.01, Fig. 5e) without decreasing P300 recruitment. This decrease was associated with a decrease in serine 5-phosphorylated RNA polymerase II recruitment to the TSS (60% decrease *p* < 0.001, Fig. 5e).

Altogether, these results demonstrate that ERα binding to the α enhancer leads to epigenetic activation of the α enhancer and 1G promoter leading to *Nr5a1* expression in immature gonadotropes.

## Discussion

Gonadotrope differentiation is a stepwise process taking place during pituitary development. Gonadotrope lineage is characterized by the expression of *Nr5a1*, a mandatory TF for gonadotrope cell identity and maturation [6]. However, the molecular mechanisms triggering *Nr5a1* expression are still poorly understood. In the current study, we have analyzed the epigenetic mechanisms implicated in this process. Using three cell lines recapitulating different stages of gonadotrope differentiation and combining chromatin accessibility analyses with studies of epigenetic mark deposition and *cis*-regulatory activity, we re-evaluated the implication of previously described *Nr5a1 cis*-regulatory sequences and characterized a yet unidentified enhancer element that initiates *Nr5a1* expression at the earliest step of gonadotrope specification.

*Nr5a1* expression has been suggested to be under the control of the 1G promoter and β enhancer in differentiating gonadotropes [8, 13]. Here, we confirmed that these two regulatory sequences are indeed activated in mature LβT2 cells. However, we showed that although the *Nr5a1* 1G promoter is already active in immature gonadotropes, this is not true for the β enhancer that does not display any active chromatin marks or *cis*-regulatory activity at this differentiation stage. This demonstrates that, in immature gonadotropes, molecular mechanisms necessary for β enhancer activity are not yet at work. The β enhancer is thus not the earliest enhancer triggering *Nr5a1* expression during gonadotrope specification.

Previous experiments performed by Stallings et al. [9] demonstrated that genomic fragments encompassing the 1G promoter are not sufficient to induce *Nr5a1* expression in vivo in the pituitary. We thus decided to analyze chromatin accessibility in the *Nr5a1* locus using our cellular models of gonadotrope differentiation to search for new *cis*-regulatory elements. Among the different potential *cis*-regulatory elements showing chromatin accessibility, one enhancer had already been characterized as a regulator of *Nr5a1* expression in fetal Leydig cells [12]. We showed that this FL enhancer is active in immature and mature cells and repressed in progenitors. It, however, cannot be implicated in the initiation of *Nr5a1* expression as it is located inside the genomic fragments that were tested as negative by Stallings et al. [9]. Interestingly, we identified a previously undescribed enhancer, the α enhancer, with an intriguing activation pattern: It is transiently and specifically activated at the immature stage in vitro. In order to investigate the activation of the α region in vivo, we studied chromatin accessibility in the *Nr5a1* locus during mouse pituitary development. Interestingly, the chromatin at the α region was only transiently accessible at E13.5 corresponding to gonadotrope cell emergence and initiation of *Nr5a1* expression. Moreover, this α enhancer displays *bonafide* active enhancer epigenetic mark decorations, with P300 recruitment, H3K27ac deposition and demethylated CpGs. Using both deletion and functional assays, we demonstrated that this enhancer is mandatory for *Nr5a1* expression in immature gonadotropes, contrary to the FL and β enhancers. Altogether, these data demonstrate that the α enhancer is the earliest activated *cis*-regulatory sequence of *Nr5a1* gene, and as such, the earliest *cis*-regulatory sequence specifically activated during gonadotrope specification.

According to our work, *Nr5a1* expression would be dynamically regulated by the sequential recruitment of two enhancers, the α enhancer during the early steps of lineage specification and then the β enhancer in mature gonadotropes. The FL enhancer, which is active during both stages, might act as a relay during this process. Supporting this hypothesis, a recent work has demonstrated the involvement of such transiently activated enhancers during the motor neuron differentiation process [19].

Analysis of the sequence conservation of the α enhancer in mammals allowed us to identify an almost perfectly conserved ERE motif. We demonstrated that ERα binds to this ERE and that regulation of the α enhancer activity is estrogen dependent. During mouse embryogenesis, *Esr1* is expressed in the developing pituitary from E12.5 onwards [data from GenePaint.org (image C1253.3.4.B) and Additional file 6A]. However, fetal circulating estrogens are believed to be inactive. Yet, active estrogens can be locally produced either by desulfonation of circulating estrogens sulfate by the steroid sulfatase (STS) enzyme or by aromatization of circulating androgens by aromatase. Genes encoding both enzymes are expressed in mouse and rat adult pituitaries [20–22]. Moreover, *Sts* transcripts (but not of the aromatase coding gene *Cyp19a1*) are expressed in progenitor and immature gonadotrope cells (Additional file 6B), supporting the idea that differentiating gonadotropes could locally produce the estrogens needed for α enhancer activation.

**Fig. 6:**
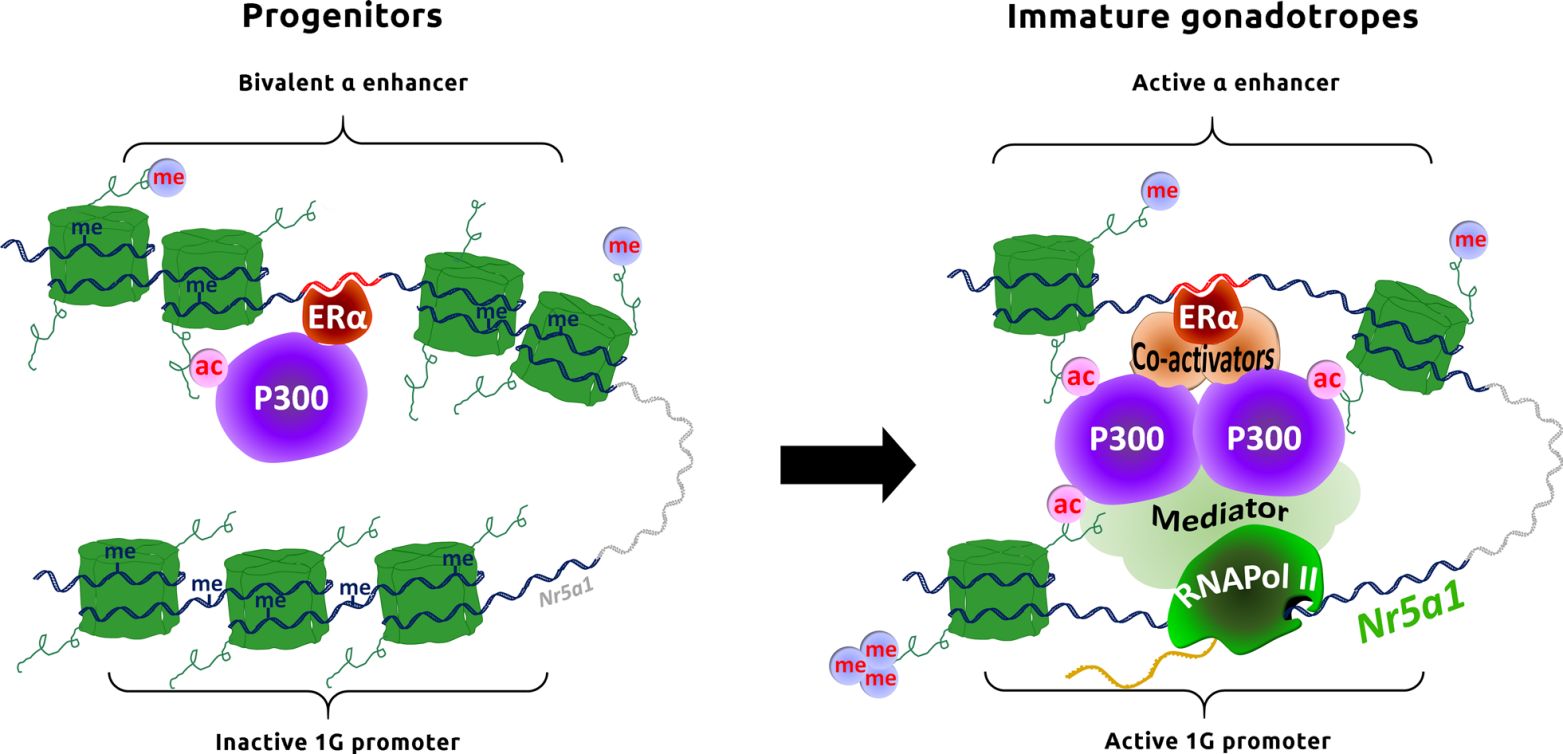
Schematic representation of the epigenetic mechanisms triggering *Nr5a1* expression during the early steps of gonadotrope specification. In progenitors, the chromatin of the *Nr5a1* locus is mainly in a repressed conformation: It is not yet accessible to TFs, DNA is methylated, and the gene is not expressed. While the promoter does not yet bear any epigenetic marks of activation, the α enhancer is already primed. Interestingly, the α enhancer is already looping on the pituitary 1G promoter and a limited chromatin accessibility of the α enhancer allows some binding of ligand-activated ERα. ERα recruits only a limited amount of P300 on the enhancer, allowing a low level of histone acetylation. The α enhancer is thus in a bivalent state and the gene is on the verge of activation. In immature gonadotropes, CpGs are demethylated and chromatin accessibility on the α enhancer increased dramatically, as well as P300 recruitment and histone acetylation. These epigenetic modifications are ERα dependent. Activation of the α enhancer allows histone acetylation and recruitment of RNA Pol II on the pituitary 1G promoter and hence *Nr5a1* transcription. The transition between α enhancer-repressed and activated states is probably due in part to recruitment of ERα co-activators that would be repressed or not yet expressed in progenitors

Interestingly, *Nr5a1* expression has been shown to be regulated by estrogens in pituitary and testis [23, 24]. In addition, pituitary *Nr5a1* expression can be affected by prenatal exposure to estrogenic endocrine-disrupting chemicals [25]. However, no functional ERE motif was found in *Nr5a1* promoters. The α enhancer is thus a *bonafide* candidate to mediate estrogen regulation of *Nr5a1* expression though ERα recruitment.

Here, by precisely deleting ERE motif from the α enhancer genomic sequence using CRISPR/Cas9 strategy, we successfully inhibited ERα binding. We observed that ERα binding is mandatory for *Nr5a1* expression in immature gonadotropes. We further demonstrated that ERα binding initiates epigenetic remodeling including P300 recruitment and subsequent H3K27ac deposition, maintaining both an open chromatin state and CpGs hypomethylation. Interestingly, we observed that ERα binding to the α enhancer also remotely regulates the 1G promoter activity. Data obtained using quantitative 3C assay strongly suggest that promoter activation is mediated in part by chromatin looping. Recent evidence obtained from studies in several cellular models [26–28] indicates that ERα may regulate gene expression mainly through enhancer activation. It has also been shown in breast cancer cells that ERα binding to enhancers increases P300 recruitment [29] and decreases DNA CpG methylation [30]. Our data demonstrate that ERα-dependent epigenetic regulation is also crucial during the earliest step of gonadotrope lineage specification.

Interestingly, we observed that ERα and P300 already bind to the α enhancer in progenitor cells and that CpGs around and inside the ERE motif remain hypomethylated. This suggests that ERα binding to the α enhancer in progenitors protects the ERE from de novo CpG methylation and allows enhancer pre-activation by increasing H3K27ac deposition. Very recently, ERα has been shown to be implicated in a biphasic recruitment of P300 on enhancers [31]: Binding of ERα first leads to P300 recruitment and histone acetylation, promoting initial enhancer pre-activation; then, ERα co-activators are recruited to reinforce P300 binding, leading to enhancer maturation and full activation.

Over-expression of ERα combined to E2 treatment in the progenitor cell line is not sufficient to induce *Nr5a1* expression (data not shown), suggesting that ERα is not able to activate the endogenous α enhancer in a repressed chromatin environment. This is consistent with several works, showing that ERα requires pioneer TFs to bind to nucleosome-masked ERE sites [32]. Thus, these mandatory pioneer factors might not yet be expressed in the progenitor cells. Based on our study, we can hypothesize that the α enhancer is in a bivalent state in progenitor gonadotropes, silent yet prone to be activated by recruitment of ERα co-activators (Fig. 6). By contrast, over-expression of ERα is sufficient to re-activate the α enhancer in the mature gonadotropes. This suggests that in mature gonadotropes, the absence of ERα expression prevents α enhancer activation although the required co-activators or pioneer factors remain expressed at this stage. Moreover, as ERα is known to be expressed in adult gonadotropes, it would suggest that α enhancer might be recruited again in adulthood to regulate dynamically *Nr5a1* expression at important stages of reproductive life.

Molecular mechanisms implicated in α enhancer activation and inhibition are in current investigation and should lead to the identification of new actors of gonadotrope lineage specification and function.

## Conclusions

Gonadotrope cell specification is still not well understood, and although key TFs and signaling pathways have been identified, the molecular events implicated in the very early steps of this lineage commitment remain elusive. Deciphering the dynamic of the regulatory enhancer network during gonadotrope specification should significantly improve the understanding of this process. This knowledge is critical for efficient reprogramming of stem cells into mature gonadotropes that would have important therapeutic applications.

## Methods

### Cell cultures

The αT1–1, αT3–1 and LβT2 mouse gonadotrope cell lines (generously given by P. Mellon, University of California, La Jolla, CA) were grown in monolayer cultures using high-glucose DMEM supplemented with 10% fetal bovine serum (FBS) and 0.2% penicillin/streptomycin, at 37 °C with 5% CO_2_.

### Antibodies

Antibodies were purchased from Abcam: anti-H3K4me1 ab8895; anti-H3K4me3 ab8580; anti-H3K27ac ab4729; anti-PolII S-5-P ab5131; anti-ERα ab32063; anti-KAT3B ab19541, and from Santa Cruz: anti-GAPDH sc-25778.

### ATAC-seq

Assay for transposase-accessible chromatin with high-throughput sequencing analysis was performed as described [33]. Briefly, 50,000 nuclei from αT1–1, αT3–1 and LβT2 cell lines were transposed using Illumina Nextera Transposase. Library fragments were amplified using NEBnext PCR master mix and custom Nextera PCR primers. Sequencing was performed on a NextSeq 500 system at the ICM iGenSeq core facility from Paris. Three independent replicates were done for each line. Data analysis was performed according to Buenrostro et al. [33]. Briefly, ATAC-seq reads were mapped on mouse genome mm9 using Bowtie 2 [34] and peak calling was done using MACS2 [35]. Peaks were then tested for consistency among three independent replicates, and data visualization was done using IVG software [36].

### ATAC-qPCR

Pregnant mice of the SWISS background at 12.5, 13.5 and 14.5 days *post coitum* were purchased from Janvier Labs. Mice were killed by cerebral dislocation and embryos retrieved and anesthetized in ice-cold PBS. Developing pituitaries were dissected under magnification glasses. Six pituitaries from age-matched embryos were pooled and cells dispersed in high-glucose DMEM with 10% FBS supplemented with 1 mg/mL collagenase D and 40 U/mL DNase I for 30 min at 37 °C. Transposition, amplification and libraries were performed as described [33]. The experiment was performed three times independently in triplicates.

For each genomic region, specific enrichments were quantified by real-time PCR using LightCycler 480 Instrument (Roche Diagnostics) and Takyon No ROX SYBR master mix (Eurogentec). Specific primers are described in Additional file 7. Raw qPCR data were normalized to control region and to E12.5 stage of development. The control region, already used in [14], is located at mm9 chr11:111,296,111–111,296,224 and is a region that displays neither enrichment for histone modifications, TF binding or chromatin accessibility so far on every tested tissues or cell types according to the ENCODE data. Change in chromatin accessibility between E12.5 and E13.5 or E12.5 and E14.5 embryonic stages was compared using a Mann–Whitney test.

### DNase I hypersensitivity assay

DNase I-sensitive assay was performed as described [14]. Briefly, 50,000 nuclei were digested with 1 U RQ1 DNase I (Promega) for 5 min at 32 °C. DNA was then extracted by proteinase K treatment and phenol/chloroform extraction. DNA fragments were segregated by size by centrifugation for 24 h at 25,000 g on a 9% sucrose cushion. A 500-µL fraction representing fragments of less than 1000 bp was collected at the top of the gradient and precipitated. The experiment was performed in triplicates on two independent clones. The α enhancer sequence enrichment in the purified DNA fractions was quantified by real-time PCR. Primers are described in Additional file 7.

### Chromatin immunoprecipitation

ChIP experiments were performed as described [14]. Briefly, 20 million cells were cross-linked with 1% formaldehyde for 10 min (or 30 min for P300 ChIP) at 37 °C, and then, formaldehyde was quenched by adding glycine (125 mM final). After nuclei extraction and lysis, chromatin was sheared by five 25-s rounds of sonication at 50% setting with a Bioblock Scientific Vibra-Cell sonicator. About 50 µg of chromatin for histone chromatin epigenetic marks and 100 µg for TFs along with 5 µg of antibodies per immunoprecipitation were combined. Immunoprecipitation was carried out at 4 °C overnight using Dynabeads™ Protein G (Invitrogen, ThermoFisher Scientific). After extensive washings and elution, chromatin cross-linking was reverted by heat and DNA purified using phenol/chloroform extraction and precipitation. Each ChIP experiment was performed at least six times independently.

For each genomic region, specific enrichments were quantified by real-time PCR. Specific primers are described in Additional file 7. Raw qPCR data were normalized to chromatin inputs and control region and were then compared using ANOVA followed by Dunnett’s multiple comparison, performed independently for each cell line and each histone marks or TF after checking for normal distribution using Kolmogorov–Smirnov test.

### Luciferase reporter assay

Cloning of the *cis*-regulatory region: Genomic regions encompassing each potential *cis*-regulatory element were amplified from mouse DNA and cloned upstream from a minimal prolactin promoter (Prl) in the pGL3-basic vector (Promega) as previously described [37]. Truncated regions were obtained by PCR or fusion PCR. Primers for amplifications and truncations are described in Additional file 7.

Cell transfection: Briefly, 50,000 cells were transiently transfected in 96-well plates with 100 ng/well of pGL3 plasmids along with 5 ng/well of pRL-SV Renilla plasmid as an internal control for normalization, using Lipofectamine^®^ 2000 (ThermoFisher Scientific) according to the manufacturer’s protocol. When indicated, cells were co-transfected with either Dharmacon ON-TARGETplus non-targeting control SiRNAs (D-001810-01-05) or ON-TARGETplus *Esr1* SiRNA (LQ-058688-01) at 10 pmol/well. When indicated, 24 h after transfection, cells were treated with either vehicle, E2, ICI 182,780 or MPP dihydrochloride at the indicated concentrations in complete steroid-containing or steroid-deprived medium. At 48 h after transfection, firefly and Renilla luciferase activities were measured using the Dual-Luciferase Reporter Assay System (Promega) according to the manufacturer’s instructions. Each experiment was performed six times independently in quadruplicates. Data were normalized to Renilla and control Pluc–Prl plasmid level for each condition. Normal distribution was checked using Kolmogorov–Smirnov test, and ANOVA followed by Dunnett’s multiple comparison tests was performed independently for each cell line and each condition.

### Bisulfite conversion of genomic DNA and sequencing

DNA methylation was performed as described previously [15]. Bisulfited fragments were cloned into pGEM-T Easy vector (Promega). At least five clones per cell line were selected and sequenced to determine the state of CpG methylation. Primers used for bisulfited DNA amplification are listed in Additional file 7.

### Quantitative chromatin conformation capture

3C experiments were conducted as described [38]. Briefly, 10 million cells were cross-linked with 2% formaldehyde for 10 min at 25 °C, and formaldehyde was then quenched by adding glycine (125 mM final). After nuclei extraction, restriction using 750 U of HindIII was carried out overnight at 37 °C under gentle agitation. After enzyme inactivation, *in nucleus* ligations were performed overnight at 16 °C. Chromatin cross-linking was reverted by heat and DNA purified using phenol/chloroform extraction and precipitation. A BAC library containing all possible chimeric fragments in equal amount was generated as followed: About 10 µg of RP23 225F7 *Nr5a1* BAC (gift from Dr CT. Gross, EMBL, Italy) was restricted with HindIII, religated and used as a control PCR template. For each cell line, four independent experiments were performed in triplicates. Chimeric DNA fragments quantification was carried out using specific primers encompassing the HindIII restriction sites on both the α enhancer and tested regions by qPCR. The BAC library was used as a standard for unbiased concentration estimation. Data were normalized to the α enhancer and internal control Ci chimeric fragment level and analyzed using Kolmogorov–Smirnov test and ANOVA followed by Dunnett’s multiple comparison tests.

### CRISPR/Cas9 deletions and dCas9–LSD1 enhancer inhibition

pLV hUbC-Cas9-T2A-GFP (#53190), pLV hUbC-dCas9-T2A-GFP (#53191) and pSPgRNA (#47108) plasmids [39] were purchased from Addgene. The *Lsd1* coding sequence was amplified from pET15B-hLSD1 (gift from Dr. Y. Shi, Harvard Medical School, Boston, USA) and cloned in frame of the C terminus of dCas9 in the pLV hUbC-dCas9-T2A-GFP plasmid. The specific guide RNAs were designed using CCTop online tool [40] and cloned in pSPgRNA plasmid.

About 5 million αT3–1 cells were electroporated with the Cas9 expression vector and sgRNA plasmids at a ratio of 1.5:8.5 µg using a Neon^®^ Transfection System (Invitrogen) according to the manufacturer’s protocol (two pulses at 1500 mV for 15 ms). The empty pSPgRNA was used as control. At 48 h post-transfection, GFP-positive cells were sorted using the FACS Aria II on the PIC2 facility of the Unit of Functional and Adaptive Biology.

For enhancer deletion studies, sorted cells were plated at low density and expanded. Single colonies were tested for deletion by PCR. A minimum of two independent homozygous clones were selected.

For enhancer decommissioning assay, sorted cells were directly processed for RNA extractions. These experiments were performed in triplicates, three times independently.

### RNA extraction and mRNA quantification

Total RNA was isolated from cells using Trizol reagent according to the manufacturer’s protocol. RNAs (1 µg) were reverse transcribed with SuperScript II reverse transcriptase (Invitrogen) using random primers according to the manufacturer’s instructions. Specific primers for qPCR quantification are described in Additional file 7. For each cell line, experiments were conducted four times independently in triplicates.

### Protein extraction and western blot

Cellular proteins were extracted in Laemmli buffer and separated on a 10% SDS–PAGE. After transfer, nitrocellulose membranes were incubated with anti-ERα (1:1000) or anti-glyceraldehyde-3-phosphate dehydrogenase (GAPDH; 1:3000) antibody in Tris-buffered saline containing 0.01% Tween 20 (TBS-T) supplemented with 5% milk overnight at 4 °C. After extensive washing, blots were incubated with a horseradish peroxidase-conjugated secondary antibody (GE healthcare #NA934V) in TBS-T/5% milk for 60 min at a room temperature and then washed. Immunodetection was performed using an enhanced chemiluminescence detection system (GE Healthcare).

### Immunohistochemistry staining of ERα

E12.5, E13.5 and E14.5 mouse embryos were proceeded for paraffin-embedded classical histology and were sectioned into 5-µm thickness. The sections were mounted on positively charged slides. The slides were deparaffinized using Histolemon and re-hydrated. Epitopes retrieval was performed by incubation in 0.05% citraconic anhydride buffer (pH 7.4) at 100  °C for 15 min. Endogenous peroxidase was inhibited by incubation in 30% H_2_O_2_ for 20 min. Endogenous biotin molecules were blocked with endogenous avidin/biotin blocking kit (ab64212), and non-specific binding was blocked by incubation with 10% goat serum diluted in PBS for 1 h. Subsequently, the sections were incubated overnight in a humid chamber at RT 1 h with ERα antibody (1/200; ab32063 abcam). Sections were extensively washed and incubated for 1 h with the biotinylated anti-rabbit IgG secondary antibody (1/500; ab97049 abcam). Sections were extensively washed and incubated 1 h with HRP-conjugated streptavidin (1/1000; ab7403 abcam). The sections were finally treated with diaminobenzidine in the dark, washed, then rapidly counterstained with Mayer’s Hemalun and mounted in Eukitt medium.

## Additional files

**Additional file 1.** Differential chromatin accessibility in gonadotropes expressed *gene* locus during specification. Chromatin accessibility was investigated by assay for transposase-accessible chromatin with high-throughput sequencing (ATAC-seq) in αT1–1, αT3–1 and LβT2 gonadotrope cell lines. ATAC-seq tracks are shown for *Isl1*, *Cga*, *Gnrhr* and *Lhb* loci. Accessible chromatin regions identified from ATAC-seq results are shown for each cell line under each track (respectively, in gray, blue and yellow). In the last lane is shown genes structure (exon in blue boxes) with proximal promoters (red boxes).

**Additional file 2.** **A** The 3′ peak in *Nr5a1 α* enhancer is inactive, while the 5′ peak displays differential *cis*-regulatory activity depending on gonadotrope differentiation stage. αT1–1, αT3–1 and LβT2 cells were transiently transfected with 5′ and 3′ peaks of the α region cloned in a pGL3b luciferase reporter system containing a minimal prolactin promoter (Pluc–Prl). Relative luciferase activity was measured as indicated in “Materials and Methods.” ANOVA followed by Dunnett’s multiple comparison tests was performed independently for each cell line. Results are normalized to control Pluc–Prl plasmid and are the mean ± SEM of six independent experiments. Significant difference with the control construct: “c” *p *< 0.001. **B** Deletion of the α enhancer using CRISPR/Cas9 in immature αT3–1 cells. Genomic sequences of the α enhancer of WT and α gRNA1–gRNA3- or α gRNA2–gRNA4-deleted αT3–1 clones were amplified and sequenced. The aligned genomic sequences of WT and deleted clones are shown along with the δERE–gRNA positions. **C** Schematic representation of the α enhancer predicted transcription factor binding sites for 31 mammals species. The α enhancer 65-bp core sequences for 31 mammalian species were analyzed using *cis*BP online library (18). Predicted TFBS are represented according to the position. Only the conserved DNA based is indicated.

**Additional file 3.** **A** The α enhancer is an inactive enhancer of *Nr5a1* in mature LβT2 cells. The α and the β enhancers were decommissioned in LβT2 cells using CRISPR/dCas9 fused with the lysine-specific histone demethylase LSD1 coding sequence (dCas9–LSD1). The dCas9–LSD1 was targeted to the α enhancer genomic sequence using the α gRNA1–gRNA3 gRNA couple and to the β enhancer genomic sequence using the β gRNA1–gRNA3 gRNA couple. Untargeting control gRNA (Ctr gRNA) was used as control. The 25% highly transfected cells were retrieved using cytometry cell sorting and tested for *Nr5a1* expression by RT-qPCR. *Nr5a1* expression level was normalized to *Gapdh*. Data are the normalized mean ± SEM of three independent experiments and are compared to cells transfected with control untargeting gRNA using Student’s *t* test “b” *p *< 0.01. **B** The *cis*-regulatory activity of the α enhancer is strictly dependent on Erα expression level and E2 in mature gonadotrope cells. LβT2 cells were transiently transfected with control (Pluc–Prl), full-length α enhancer (Pluc–α enh) or the mutated α enhancer (Pluc–α enh MutERE) constructs along with psg5 ERα expression plasmid or psg5 control plasmid in a steroid-deprived medium. Transfected cells were treated with either vehicle or E2 at 1 nM. Relative luciferase activity was measured as indicated in “Materials and Methods.” Results are normalized to corresponding Pluc–Prl plasmid and are the mean ± SEM of six independent experiments. ANOVA followed by Dunnett’s multiple comparison tests was performed: significant difference with the vehicle condition (gray bar): “c” *p *< 0.001. **C** ERα expression is sufficient to activate endogenous α enhancer in mature gonadotrope cells. LβT2 cells were transiently co-transfected with control (psg5), or psg5-ERα expression plasmid and pEGFP-N1. An ATAC assay followed by real-time PCR quantification (ATAC-qPCR) was performed on the 25% highly transfected GFP cells retrieved using cytometry cell sorting. Quantitative PCR was performed using primers targeting *Nr5a1* α and β enhancers. Raw qPCR data were normalized to control region. Results are the mean ± SEM of three independent experiments. Significant difference with the control psg5 transfected condition “b” *p* < 0.01.

**Additional file 4.** **A** ERα binds to the α enhancer in progenitor αT1–1 gonadotropes. ERα binding on the α enhancer chromatin was investigated using ChIP assays in αT1–1 cells. Quantitative PCR was performed using primers targeting the α enhancer genomic sequence. Raw qPCR data were normalized to input. The final results were expressed as fold over the control region. Results are the mean ± SEM of three independent experiments in triplicates. Significant difference with the control region was analyzed using Student’s *t*-test: “b” *p *< 0.01. **B** P300 binds to α enhancer in progenitor αT1–1 gonadotropes. P300 binding on the α enhancer chromatin was investigated using ChIP assays in αT1–1 cells. Quantitative PCR was performed using primers targeting the α enhancer genomic sequence. Raw qPCR data were normalized to input. The final results were expressed as fold over the control region. Results are the mean ± SEM of three independent experiments in triplicates. Significant difference with the control region was analyzed using Student’s *t*-test: “c” *p *< 0.001. **C** The *cis*-regulatory activity of the α enhancer is dependent on *Erα* expression level in progenitor αT1–1 gonadotropes. αT1–1 cells were transiently co-transfected with control (Pluc–Prl) or full-length α enhancer (Pluc–α enh) Pluc constructs and with scramble or *Erα* SiRNA. Relative luciferase activity was measured as indicated in “Materials and Methods.” Results were normalized to control Pluc–Prl plasmid and are the mean ± SEM of three independent experiments in quadruplicates. Significant difference with the scramble SiRNA using Student’s *t*-test “c” *p* < 0.001.

**Additional file 5.** **A** Knockdown efficiency of ERα SiRNA in αT3–1 cells. αT3–1 cells were transiently transfected in duplicates with scramble or *Erα* SiRNA. Proteins were extracted 48 h later. Western blots for ERα and GAPDH immunodetection were performed as indicated in “Materials and Methods.” Top: ERα immunodetection: The 66-kDa and the 36-kDa isoforms are expressed in αT3–1 cells. *Erα* SiRNA allows efficient knockdown of both isoforms. Bottom: GAPDH immunodetection for normalization. **B** ERα specific antagonist MPP dihydrochloride modulates α enhancer *cis*-regulatory activity. αT3–1 cells were transiently transfected with control (Prl) or full-length α enhancer (α enh) Pluc constructs. Transfected cells were treated with either vehicle or MPP dihydrochloride at the indicated concentrations. Relative luciferase activity was measured as indicated in “Materials and Methods.” Results were normalized for control Pluc–Prl–luc and are the mean ± SEM of six independent experiments in quadruplicates. ANOVA followed by Dunnett’s multiple comparison tests was performed to compare drugs at different concentrations against vehicle condition. Significant difference with the vehicle: “c” *p *< 0.001.

**Additional file 6.** **A** ERα expression in the developing mouse pituitary. ERα immunohistochemistry analysis of pituitaries of embryos at E12.5, E13.5 and E14.5. ERα is expressed at E12.5, E13.5 and E14.5 in the developing pituitary. Negative controls with no ERα antibodies were performed and yielded no signal (data not shown). Magnification: 600X. **B** *Cyp19a1* and *Sts* expression during gonadotrope cell differentiation. *Cyp19a1* and *Sts* expressions in αT1–1, αT3–1 and LβT2 cells were measured by RT-qPCR. Expression level was normalized to *Gapdh*. Data are the normalized mean ± SEM of three independent experiments. Significant difference with water: “c” *p *< 0.001. Nd: not detected.

**Additional file 7.** Primers used in this study.

## Data Availability

The datasets used and analyzed during the current study are available from the corresponding author on reasonable request.
