# Supplementary figures and images for: Identification of a pituitary ERα-activated enhancer triggering the expression of Nr5a1, the earliest gonadotrope lineage-specific transcription factor
Source: Epigenetics Chromatin. 2019 Aug 7;12:48. doi: 10.1186/s13072-019-0291-8 (PMC6685283; doi:10.1186/s13072-019-0291-8)

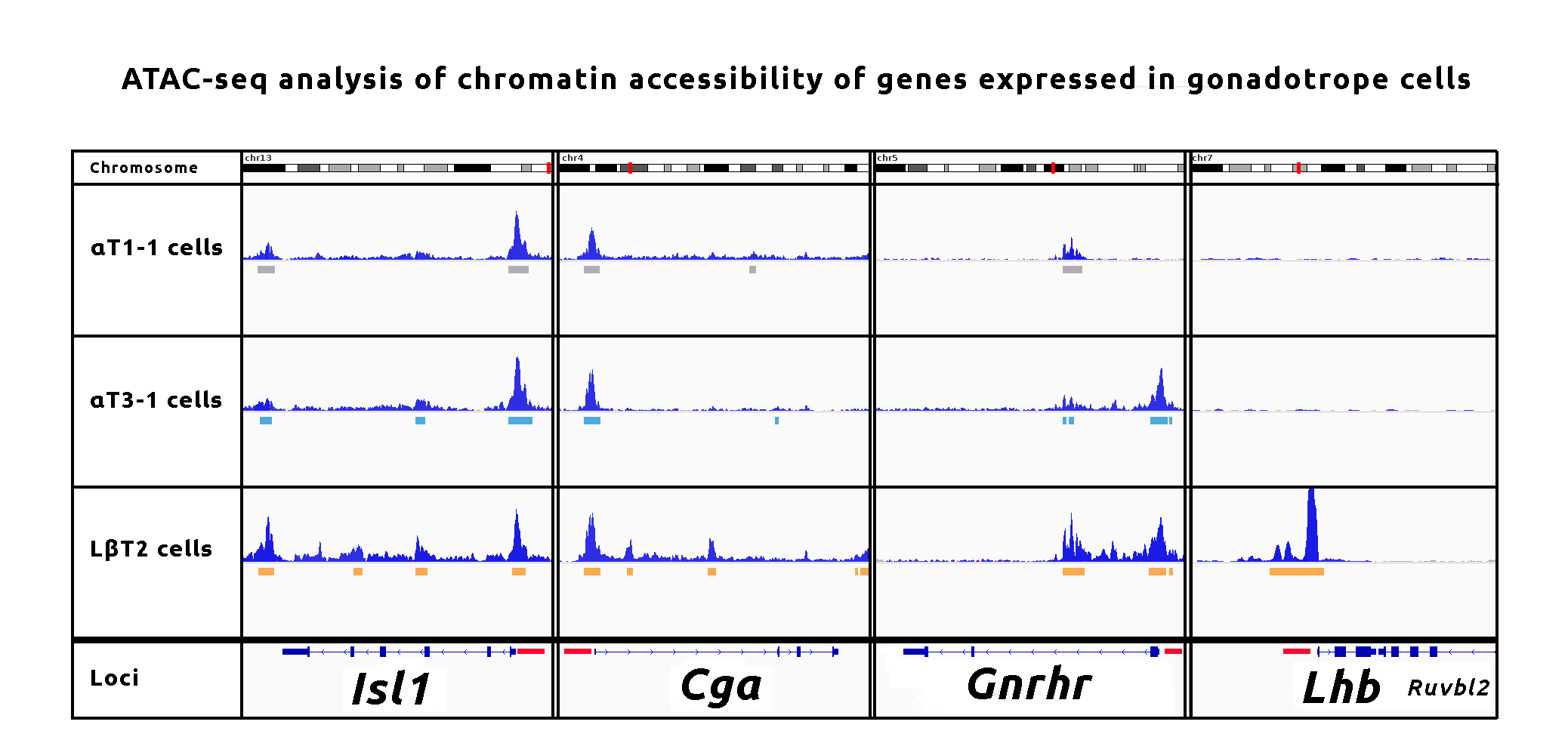

Supplement: Supplementary file 1 — Additional file 1. Differential chromatin accessibility in gonadotropes expressed gene locus during specification. Chromatin accessibility was investigated by assay for transposase-accessible chromatin with high-throughput sequencing (ATAC-seq) in αT1–1, αT3–1 and LβT2 gonadotrope cell lines. ATAC-seq tracks are shown for Isl1, Cga, Gnrhr and Lhb loci. Accessible chromatin regions identified from ATAC-seq results are shown for each cell line under each track (respectively, in gray, blue and yellow). In the last lane is shown genes structure (exon in blue boxes) with proximal promoters (red boxes). [file 13072_2019_291_MOESM1_ESM.tif]

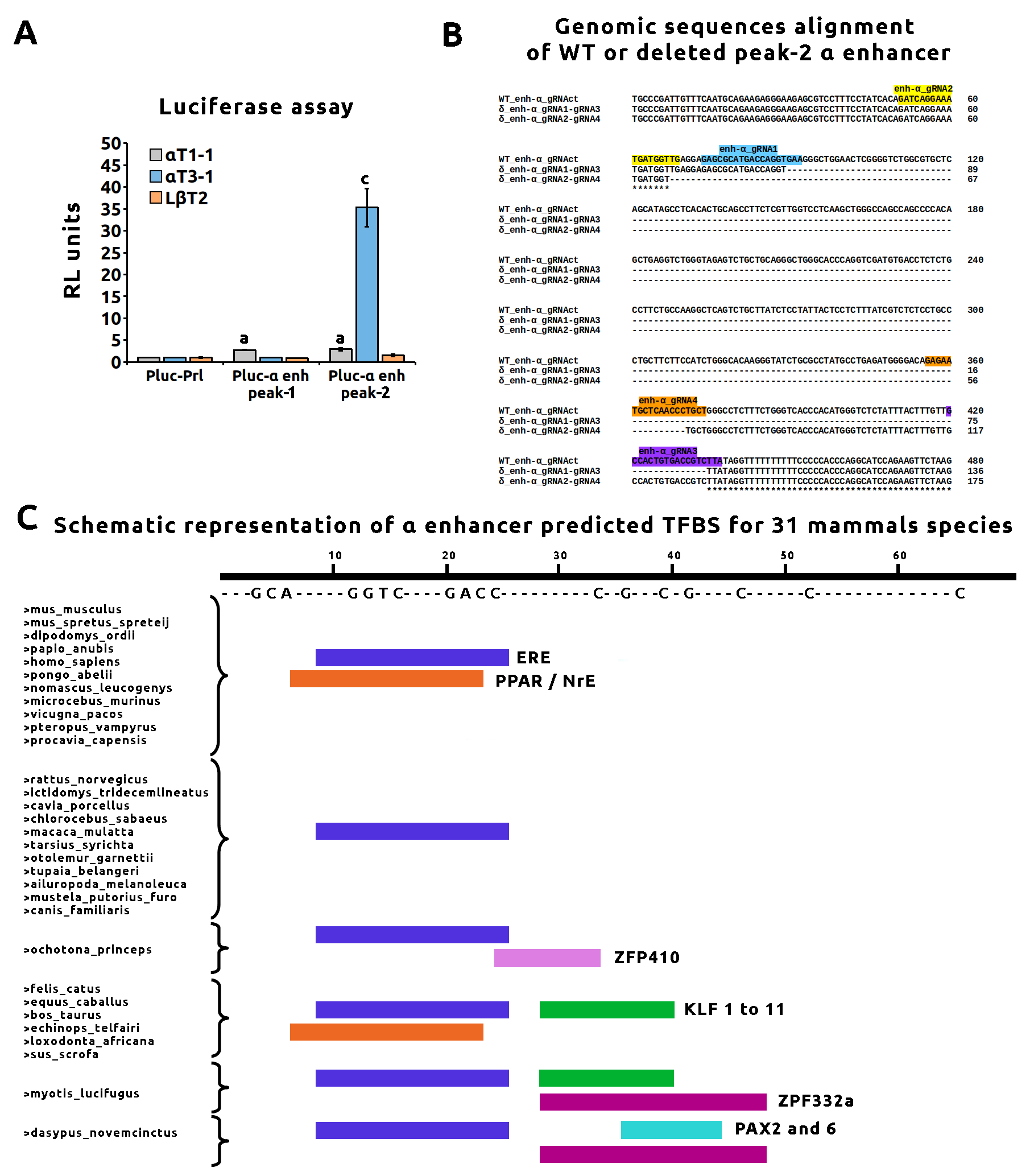

Supplement: Supplementary file 2 — Additional file 2. A The 3′ peak in Nr5a1 α enhancer is inactive, while the 5′ peak displays differential cis-regulatory activity depending on gonadotrope differentiation stage. αT1–1, αT3–1 and LβT2 cells were transiently transfected with 5′ and 3′ peaks of the α region cloned in a pGL3b luciferase reporter system containing a minimal prolactin promoter (Pluc–Prl). Relative luciferase activity was measured as indicated in “Materials and Methods.” ANOVA followed by Dunnett’s multiple comparison tests was performed independently for each cell line. Results are normalized to control Pluc–Prl plasmid and are the mean ± SEM of six independent experiments. Significant difference with the control construct: “c” p < 0.001. B Deletion of the α enhancer using CRISPR/Cas9 in immature αT3–1 cells. Genomic sequences of the α enhancer of WT and α gRNA1–gRNA3- or α gRNA2–gRNA4-deleted αT3–1 clones were amplified and sequenced. The aligned genomic sequences of WT and deleted clones are shown along with the δERE–gRNA positions. C Schematic representation of the α enhancer predicted transcription factor binding sites for 31 mammals species. The α enhancer 65-bp core sequences for 31 mammalian species were analyzed using cisBP online library (18). Predicted TFBS are represented according to the position. Only the conserved DNA based is indicated. [file 13072_2019_291_MOESM2_ESM.tif]

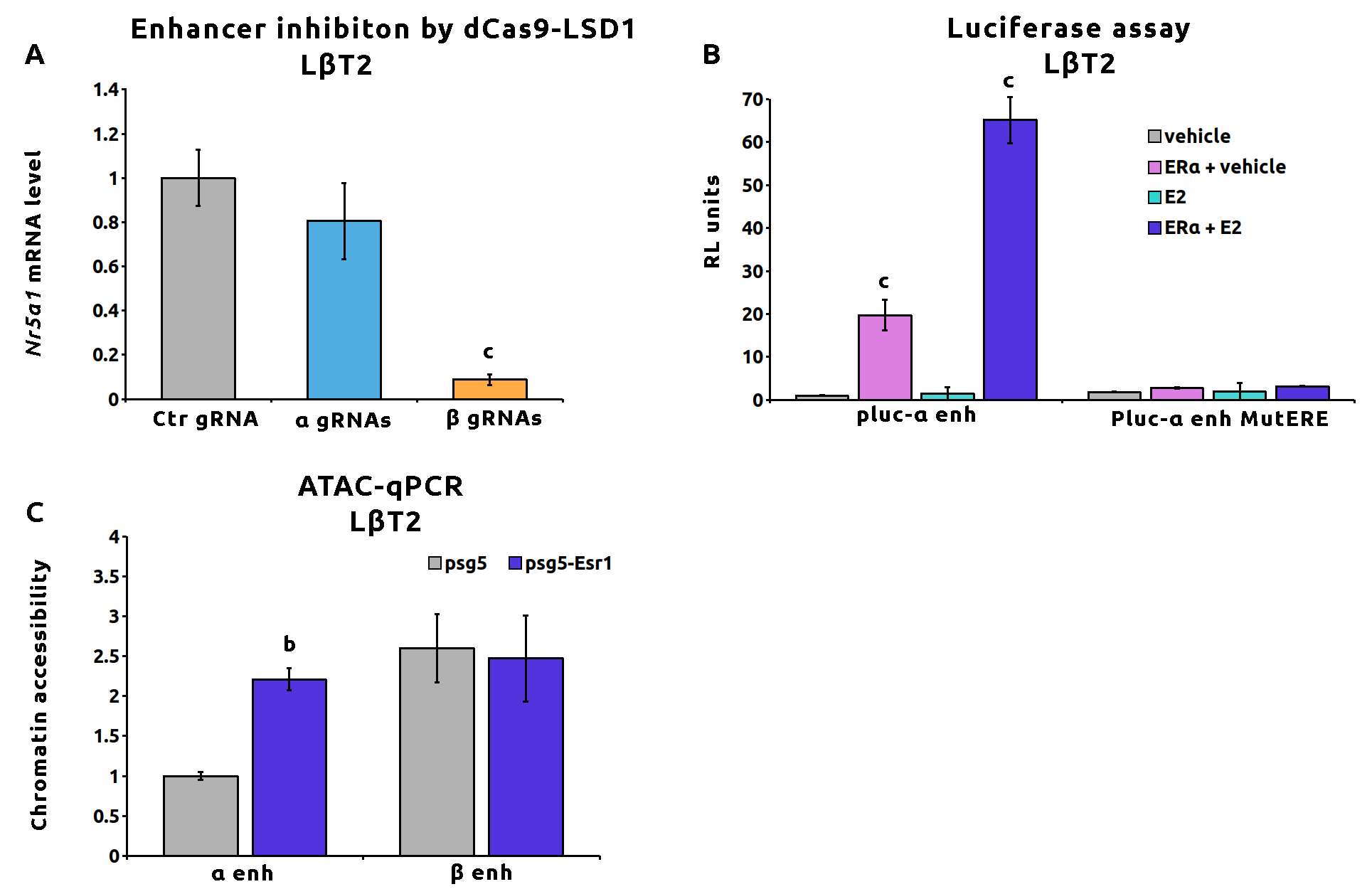

Supplement: Supplementary file 3 — Additional file 3. A The α enhancer is an inactive enhancer of Nr5a1 in mature LβT2 cells. The α and the β enhancers were decommissioned in LβT2 cells using CRISPR/dCas9 fused with the lysine-specific histone demethylase LSD1 coding sequence (dCas9–LSD1). The dCas9–LSD1 was targeted to the α enhancer genomic sequence using the α gRNA1–gRNA3 gRNA couple and to the β enhancer genomic sequence using the β gRNA1–gRNA3 gRNA couple. Untargeting control gRNA (Ctr gRNA) was used as control. The 25% highly transfected cells were retrieved using cytometry cell sorting and tested for Nr5a1 expression by RT-qPCR. Nr5a1 expression level was normalized to Gapdh. Data are the normalized mean ± SEM of three independent experiments and are compared to cells transfected with control untargeting gRNA using Student’s t test “b” p < 0.01. B The cis-regulatory activity of the α enhancer is strictly dependent on Erα expression level and E2 in mature gonadotrope cells. LβT2 cells were transiently transfected with control (Pluc–Prl), full-length α enhancer (Pluc–α enh) or the mutated α enhancer (Pluc–α enh MutERE) constructs along with psg5 ERα expression plasmid or psg5 control plasmid in a steroid-deprived medium. Transfected cells were treated with either vehicle or E2 at 1 nM. Relative luciferase activity was measured as indicated in “Materials and Methods.” Results are normalized to corresponding Pluc–Prl plasmid and are the mean ± SEM of six independent experiments. ANOVA followed by Dunnett’s multiple comparison tests was performed: significant difference with the vehicle condition (gray bar): “c” p < 0.001. C ERα expression is sufficient to activate endogenous α enhancer in mature gonadotrope cells. LβT2 cells were transiently co-transfected with control (psg5), or psg5-ERα expression plasmid and pEGFP-N1. An ATAC assay followed by real-time PCR quantification (ATAC-qPCR) was performed on the 25% highly transfected GFP cells retrieved using cytometry cell sorting. Quantitative PCR w [file 13072_2019_291_MOESM3_ESM.tif]

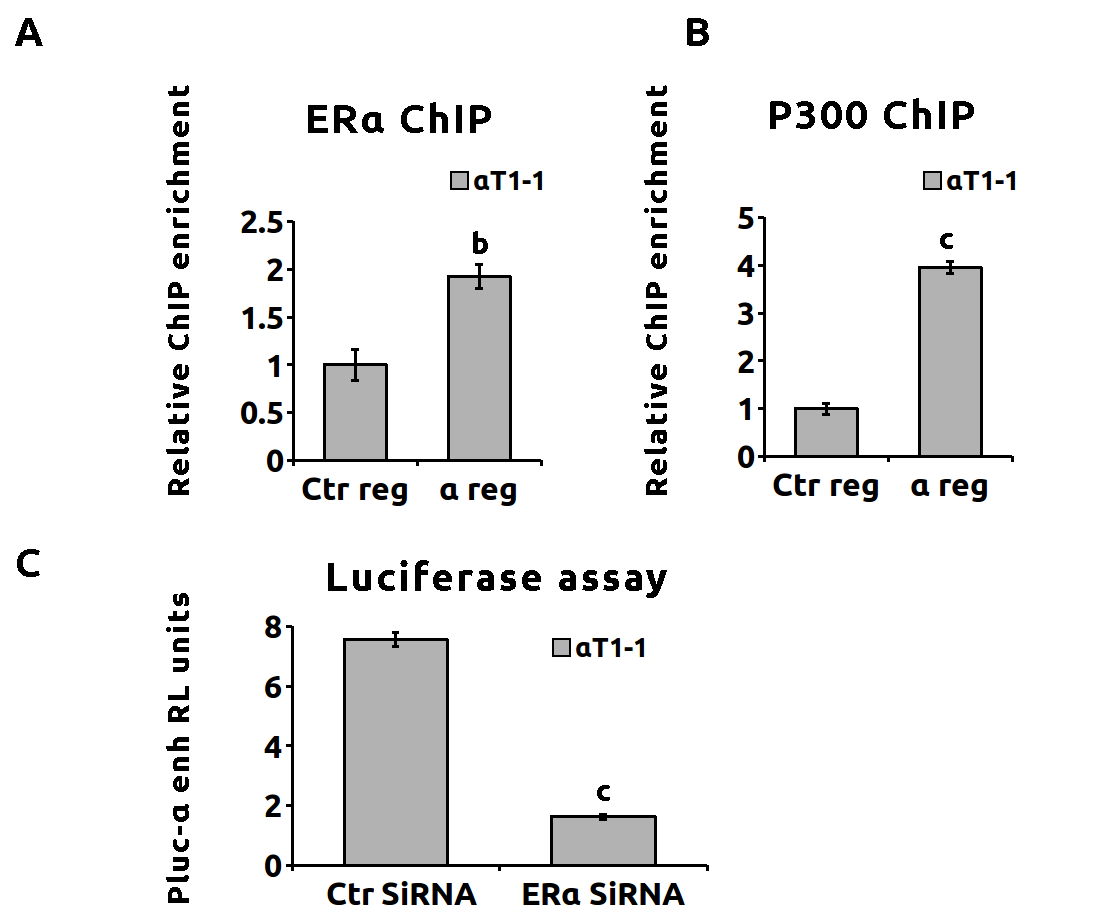

Supplement: Supplementary file 4 — Additional file 4. A ERα binds to the α enhancer in progenitor αT1–1 gonadotropes. ERα binding on the α enhancer chromatin was investigated using ChIP assays in αT1–1 cells. Quantitative PCR was performed using primers targeting the α enhancer genomic sequence. Raw qPCR data were normalized to input. The final results were expressed as fold over the control region. Results are the mean ± SEM of three independent experiments in triplicates. Significant difference with the control region was analyzed using Student’s t-test: “b” p < 0.01. B P300 binds to α enhancer in progenitor αT1–1 gonadotropes. P300 binding on the α enhancer chromatin was investigated using ChIP assays in αT1–1 cells. Quantitative PCR was performed using primers targeting the α enhancer genomic sequence. Raw qPCR data were normalized to input. The final results were expressed as fold over the control region. Results are the mean ± SEM of three independent experiments in triplicates. Significant difference with the control region was analyzed using Student’s t-test: “c” p < 0.001. C The cis-regulatory activity of the α enhancer is dependent on Erα expression level in progenitor αT1–1 gonadotropes. αT1–1 cells were transiently co-transfected with control (Pluc–Prl) or full-length α enhancer (Pluc–α enh) Pluc constructs and with scramble or Erα SiRNA. Relative luciferase activity was measured as indicated in “Materials and Methods.” Results were normalized to control Pluc–Prl plasmid and are the mean ± SEM of three independent experiments in quadruplicates. Significant difference with the scramble SiRNA using Student’s t-test “c” p < 0.001. [file 13072_2019_291_MOESM4_ESM.tif]

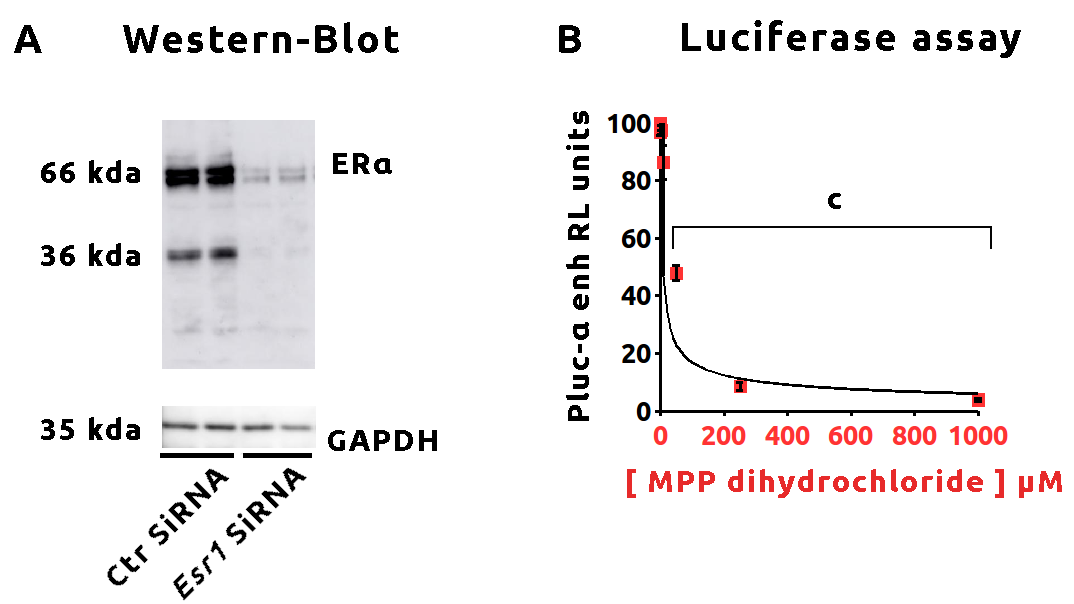

Supplement: Supplementary file 5 — Additional file 5. A Knockdown efficiency of ERα SiRNA in αT3–1 cells. αT3–1 cells were transiently transfected in duplicates with scramble or Erα SiRNA. Proteins were extracted 48 h later. Western blots for ERα and GAPDH immunodetection were performed as indicated in “Materials and Methods.” Top: ERα immunodetection: The 66-kDa and the 36-kDa isoforms are expressed in αT3–1 cells. Erα SiRNA allows efficient knockdown of both isoforms. Bottom: GAPDH immunodetection for normalization. B ERα specific antagonist MPP dihydrochloride modulates α enhancer cis-regulatory activity. αT3–1 cells were transiently transfected with control (Prl) or full-length α enhancer (α enh) Pluc constructs. Transfected cells were treated with either vehicle or MPP dihydrochloride at the indicated concentrations. Relative luciferase activity was measured as indicated in “Materials and Methods.” Results were normalized for control Pluc–Prl–luc and are the mean ± SEM of six independent experiments in quadruplicates. ANOVA followed by Dunnett’s multiple comparison tests was performed to compare drugs at different concentrations against vehicle condition. Significant difference with the vehicle: “c” p < 0.001. [file 13072_2019_291_MOESM5_ESM.tif]

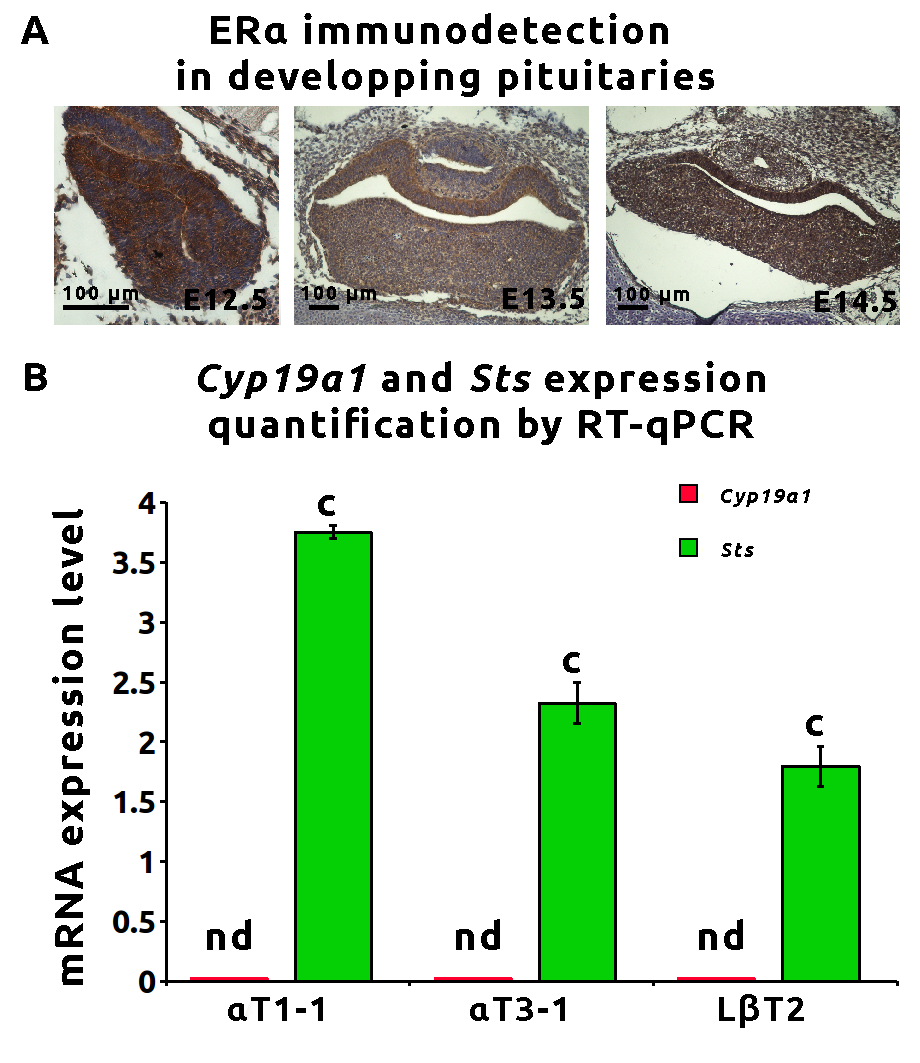

Supplement: Supplementary file 6 — Additional file 6. A ERα expression in the developing mouse pituitary. ERα immunohistochemistry analysis of pituitaries of embryos at E12.5, E13.5 and E14.5. ERα is expressed at E12.5, E13.5 and E14.5 in the developing pituitary. Negative controls with no ERα antibodies were performed and yielded no signal (data not shown). Magnification: 600X. B Cyp19a1 and Sts expression during gonadotrope cell differentiation. Cyp19a1 and Sts expressions in αT1–1, αT3–1 and LβT2 cells were measured by RT-qPCR. Expression level was normalized to Gapdh. Data are the normalized mean ± SEM of three independent experiments. Significant difference with water: “c” p < 0.001. Nd: not detected. [file 13072_2019_291_MOESM6_ESM.tif]
